# Combining Gene Signatures Improves Prediction of Breast Cancer Survival

**DOI:** 10.1371/journal.pone.0017845

**Published:** 2011-03-10

**Authors:** Xi Zhao, Einar Andreas Rødland, Therese Sørlie, Bjørn Naume, Anita Langerød, Arnoldo Frigessi, Vessela N. Kristensen, Anne-Lise Børresen-Dale, Ole Christian Lingjærde

**Affiliations:** 1 Department of Genetics, Institute for Cancer Research, Oslo University Hospital, Radiumhospitalet, Oslo, Norway; 2 Institute of Clinical Medicine, University of Oslo, Oslo, Norway; 3 Biomedical Research Group, Department of Informatics, Faculty of Mathematics and Natural Sciences, University of Oslo, Oslo, Norway; 4 Center for Cancer Biomedicine, University of Oslo, Oslo, Norway; 5 Cancer Clinic, Oslo University Hospital, Oslo, Norway; 6 Department of Biostatistics, Institute of Basic Medical Sciences, University of Oslo, Oslo, Norway; 7 Norwegian Computing Center, Oslo, Norway; National Cancer Institute, United States of America

## Abstract

**Background:**

Several gene sets for prediction of breast cancer survival have been derived from whole-genome mRNA expression profiles. Here, we develop a statistical framework to explore whether combination of the information from such sets may improve prediction of recurrence and breast cancer specific death in early-stage breast cancers. Microarray data from two clinically similar cohorts of breast cancer patients are used as training (n = 123) and test set (n = 81), respectively. Gene sets from eleven previously published gene signatures are included in the study.

**Principal Findings:**

To investigate the relationship between breast cancer survival and gene expression on a particular gene set, a Cox proportional hazards model is applied using partial likelihood regression with an L2 penalty to avoid overfitting and using cross-validation to determine the penalty weight. The fitted models are applied to an independent test set to obtain a predicted risk for each individual and each gene set. Hierarchical clustering of the test individuals on the basis of the vector of predicted risks results in two clusters with distinct clinical characteristics in terms of the distribution of molecular subtypes, ER, PR status, *TP53* mutation status and histological grade category, and associated with significantly different survival probabilities (recurrence: *p* = 0.005; breast cancer death: *p* = 0.014). Finally, principal components analysis of the gene signatures is used to derive combined predictors used to fit a new Cox model. This model classifies test individuals into two risk groups with distinct survival characteristics (recurrence: *p* = 0.003; breast cancer death: *p* = 0.001). The latter classifier outperforms all the individual gene signatures, as well as Cox models based on traditional clinical parameters and the Adjuvant! Online for survival prediction.

**Conclusion:**

Combining the predictive strength of multiple gene signatures improves prediction of breast cancer survival. The presented methodology is broadly applicable to breast cancer risk assessment using any new identified gene set.

## Introduction

Cancer is a complex disease characterized by a number of genetic and epigenetic abnormalities. Patients associated with similar clinical and pathological parameters may have very different tumor profiles at the molecular level and may respond differently to treatment [1], [2], [3], [4]. Genome-wide expression profiling of tumors has become an important tool to identify gene sets and gene signatures that can be used to predict clinical endpoints, such as survival and therapy response [1], [2], [3], [5], [6], [7], [8], [9], [10], [11], [12]. A number of tumor classification algorithms based on gene expression profiles have been proposed, using clinical data or known biological class labels to build predictive models for outcome: e.g. the 70-gene signature MammaPrint® [3], the 76-gene signature of Wang et al. [8] and the Genomic Grade Index [4]. Several classification methods also utilize unsupervised methods to discover biological subgroups [1], [5], [9].

Published gene signatures that are predictive of clinical outcome in breast cancer are partly or completely based on different genes. Nevertheless, their predictions are often in good concordance in terms of assigning new patient samples into groups of poor and good outcome [13], [14]. This indicates that some common biological processes overlap across those gene signatures [14], [15], but the reported gene signatures are likely to capture various biological aspects of breast cancer. Hence, by combining information from multiple gene signatures, one would potentially increase the prediction power and bring out an overall picture of this disease.

We therefore aim to develop an analytical framework that allows us to utilize the combined strength from individual gene signatures. Such a framework and the resulting model will be broadly applicable for survival prediction across heterogeneous tumor groups capturing a broad spectrum of biological aspects.

Using the original gene signatures would generally require recalibration on new datasets. As a consequence, the original gene signatures will not always be maintained and used as originally intended. In this paper, the aim is not to compare or condition on the original gene signatures per se, but rather to focus on the genes themselves. Thus we rely only on the gene sets on which the gene signatures are based, and fit survival models based on these gene sets without depending on the quantitative specifics of the published gene signatures. In doing so, we are able to utilize what is arguably the most critical piece of information from the gene signatures, and analytically most challenging to derive: shortlists of genes related to breast cancer survival. It should be emphasized that the main aim of our study is to present a method for explorative analyses and for seeding gene selection algorithms with prior known gene sets, rather than a claimed method for producing optimized gene signatures competing with published counterparts.

**Figure 1:**
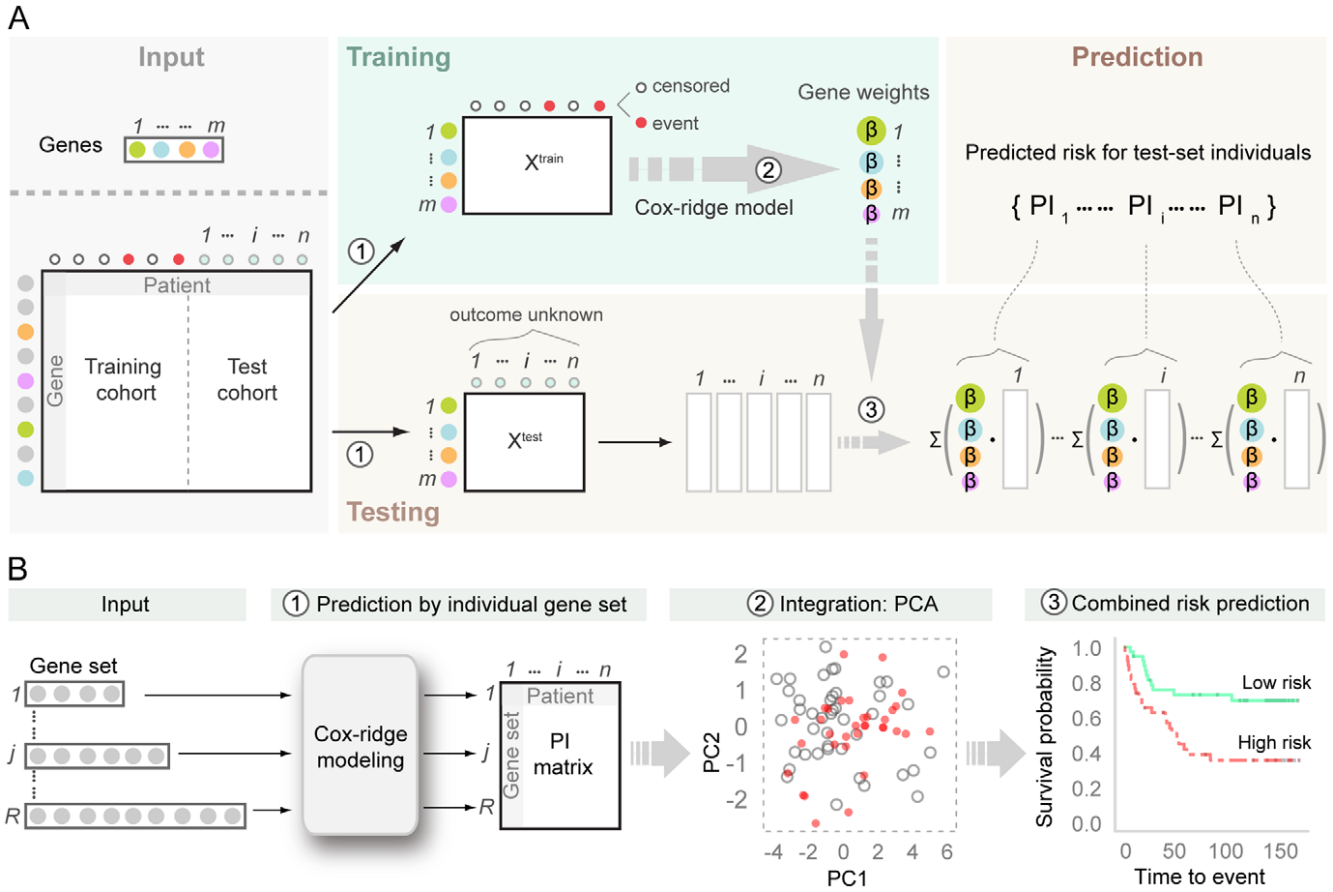
Flowchart of the analysis. (**A**) Construction of the gene-set predictor/gene signature for risk prediction. Input: A set of genes of interest (gene *1*, …, *m*) which can be traced by the corresponding colors through out the diagram; gene expression data for training cohort and test cohort with genes placed in the rows and patients in the columns. *Step 1*. Gene identity mapping and extract expression matrix. *Step 2*. With available status of observing an event for the patients on the training set, a Cox model with L2 penalty is used to model the relationship of survival probability and gene expression pattern of the gene set. The coefficients or “gene weights” (*β_1_, …, β_m_*) associated with individual genes are estimated from the Cox-ridge model. Size of the bubble in the gene weights matrix reflects the importance of the corresponding gene for survival prediction. *Step 3*. A *Prognostic Index* (PI), the predicted risk score for a test patient *i* (*i = 1, …, n*) is calculated by the sum of weighted gene expression from test patient *i* using the estimated gene weights from step2. (**B**) Integration of multiple gene signatures by dimension reduction. Input multiple gene sets of interest together with their gene expression data. *Module 1*: For *j*th gene set (*j* = 1, *…, R*), the procedure described in panel A is used to predict a risk score PI for individual test patient. The resulting PI matrix is positioned in *R* by *n* dimension representing the risk prediction of the *n* test patients by each of the *R* gene sets. *Module 2:* Integrate predictions from multiple gene signatures by dimension reduction using principal components analysis (PCA). *Module 3:* Dichotomize the risk scores on PC1 by median (higher than median indicates high risk) resulting in two predicted risk groups for survival outcome.

We use the gene sets of eleven published gene signatures to analyze breast cancer survival and relapse (Table 1). We aim to build survival predictors on a common data set to achieve a fair comparison of the gene sets. For each gene set, we fit survival models to one dataset and apply them to another to obtain predictions of cancer relapse and death, resulting in a predictive index (PI) per patient. We then combine the PIs obtained for a patient by extracting a common signal using the first principal component, in order to improve the predictions obtained by each gene set separately. We illustrate the capacity of the proposed framework to lead to improved survival prediction. A flowchart of the analysis is shown in Figure 1; see specific sections under Results for a detailed explanation of the different parts of the figure.

**Table 1:** Published gene sets included in the analysis.

Coding	Full name	Platform
RS	16-gene-recurrence-score predictor [6]	Oncotype DX assay
SD	26-gene stroma-derived prognostic predictor [21]	Agilent Human oligo microarrays
LM	54-lung-metastasis-gene signature [7]	Affymetrix GeneChips
AMST	70-gene predictor [3]	Agilent Human oligo microarrays
ROT	76-gene predictor [8]	Affymetrix GeneChips
Grade	97- histologic-grade-associated markers [4]	Affymetrix GeneChips
Robust	127-gene classifier [31]	Affymetrix GeneChips
Hypoxia	168-hypoxia-gene signature [9]	Stanford 43k cDNA array
Stem	186-invasiveness-gene signature [10]	Affymetrix GeneChips
Intrinsic	306-intrinsic/UNC gene list [12]	Agilent Human oligo microarrays
WR	512 wound response gene list [5]	Stanford 43k cDNA array

## Results

### Cross-platform gene mapping

Using the gene mapping procedure described in Methods, we were able to identify and map at least 80% of the genes from each of the originally published gene sets to the Stanford 43k cDNA array. Table 2 summarizes the number of genes mapped to the training set (MicMa [11]) and test set (Ull [16]). The number of genes that overlap between the different gene sets is shown in Table 3. The percentage of overlap ranges from 0 to 25% (between Robust and Grade) with a median overlap of 0.57%.

**Table 2:** Acronyms for original gene sets and coverage on training & test set.

Gene set	Mapped genes #	Coverage %
RS	15	15/16 = 94%
SD	22	22/26 = 85%
LM	48	48/54 = 89%
AMST	57	57/70 = 81%
ROT	66	66/76 = 87%
Grade	111	111/128§ = 87%
Robust	114	114/127 = 90%
Hypoxia	168	168/168 = 100%
Stem	161	161/186 = 87%
Intrinsic	290	290/306 = 95%
WR	561	561/573‡ = 98%

§ 128 Affymetrix probe IDs were used instead of 97 gene symbols.

‡ 573 image clone IDs were used instead of 512 genes.

**Table 3:** Number of overlapping genes between gene sets.

	RS	SD	LM	AMST	ROT	Grade	Robust	Hypoxia	Stem	Intrinsic	WR
RS		0	0	1	0	3	2	0	1	5	1
SD	0		0	1	0	0	1	1	0	0	1
LM	0	0		0	0	0	0	1	0	7	5
AMST	1	1	0		0	7	7	1	0	2	3
ROT	0	0	0	0§		7	2	1	0	4	3
Grade	3	0	0	7	7		45	0	0	11	12
Robust	2	1	0	7	2	45		2	4	11	10
Hypoxia	0	1	1	1	1	0	2		4	3	12
Stem	1	0	0	0	0	0	4	4		5	7
Intrinsic	5	0	7	2	4	11	11	3	5		19
WR	1	1	5	3	3	12	10	12	7	19	

§ There was 1 gene overlapping between ROT & AMST according to the previous report [15]: *CCNE2* (GenBankID NM_004702). However, in the newer version of the NCBI database: “NM_004702.2 was permanently suppressed because the transcript has insufficient support and is a nonsense-mediated mRNA decay (NMD) candidate.” (http://www.ncbi.nlm.nih.gov/entrez/viewer.fcgi?db=nuccore&id=17318566. Accessed on Mar. 18, 2009).

### Penalized Cox regression

For each gene set, a Cox model was used to relate time to systemic relapse to gene expression (see the second last section in Results for analysis using breast cancer specific death as the clinical endpoint). A weight is thus assigned to each gene and a risk score (or *prognostic index, PI*) is found for each individual by adding together the weighted gene expressions. Due to the large number of covariates, ranging from 15 genes (RS; [6]) up to 561 genes (WR; [5]), we estimated a penalized partial likelihood, where the penalty is proportional to the Euclidean (L2) norm of the parameter vector. This has the effect of avoid over-fitting by reducing the variance of the estimator, at the cost of introducing a bias. A penalty parameter *λ* controls the trade-off between the goodness of fit (imposed by the partial likelihood) and low variance (imposed by the penalty term). As *λ*→0 the solution approaches that of ordinary Cox regression, while as *λ*→∞ the solution approaches that of a constant estimator that does not depend on the expression of any gene. In practice, a good choice for *λ* has to be determined empirically, and for this purpose we applied leave-one-out cross-validation (LOOCV) [17]. The cross-validation curves obtained for each of the gene sets are shown in Figure S1, and the *λ* that maximizes the cross-validation function can be found in Table 4. For gene sets *SD* and *LM*, the cross-validation function did not have a maximum in the search range resulting in a null model that has no covariates.

**Table 4:** Individual gene set prediction characteristics (optimal *λ* by LOOCV in model building, change in deviance on test set, standard deviation for PIs).

	Prediction characteristics statistic		
Gene Set	*λ*_opt_	Δ Deviance	Sd (PI)
RS	338	-1.4	0.109
SD	-	0	0
LM	-	0	0
AMST	227	0.3	0.195
ROT	1029	0.1	0.095
Grade	3580	-2.12	0.078
Robust	1705	-2.57	0.121
Hypoxia	392	-0.71	0.245
Stem	3623	-0.25	0.044
Intrinsic	1576	-0.88	0.137
WR	11261	-0.37	0.037

### Prediction of survival with the prognostic index

**Figure 2:**
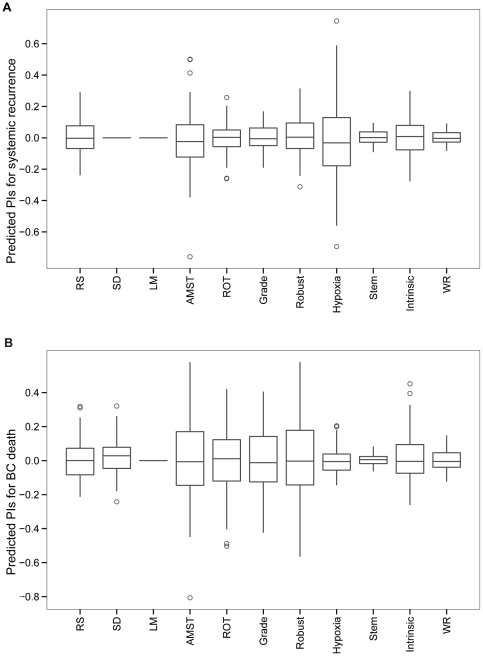
Boxplot of predicted PIs on test data. (**A**) Systemic recurrence: The figure shows that the predicted PIs across all the studied gene sets were roughly centered around 0, resulting from the standardization procedure of the expression matrix on both training and test set for individual gene set in the model building stage. The standard deviations of PIs for individual gene set are following: RS: 0.109; SD: 0; LM: 0; AMST: 0.195; ROT: 0.095; Grade: 0.078; Robust: 0.121; Hypoxia: 0.245; Stem: 0.044; Intrinsic: 0.137; WR: 0.037. Due to lack of convergence, the predicted PIs by gene set SD and LM was calculated by setting tuning parameter *λ,* at a large value. (**B**) Breast cancer specific death (BC specific death): Boxplot of predicted PIs on test set. Gene set LM failed to converge in the model training.

Adding together the weighted gene expressions for a particular gene set, each patient in the test set was assigned a Prognostic Index (PI). The distributions of the PIs are shown in Figure 2A; observe that some gene sets discriminate the patients on a wider range of risk scores than others. The deviance, an indicator of the models' goodness of fit, was calculated for each gene set. Table 4 shows the difference in deviance (ΔD) between the fitted models and a null model with no genes. The magnitude of ΔD indicated the prediction power gained by a gene-set predictor. For gene sets with positive ΔD, the corresponding gene-set models were likely to perform poorly in prediction. Since no optimal *λ* was found for gene set *SD* and *LM*, the corresponding ΔD was 0. The standard deviation of the empirical PI distributions can be found in Table 4.

### Similarities between PIs

**Figure 3 pone-0017845-g003:**
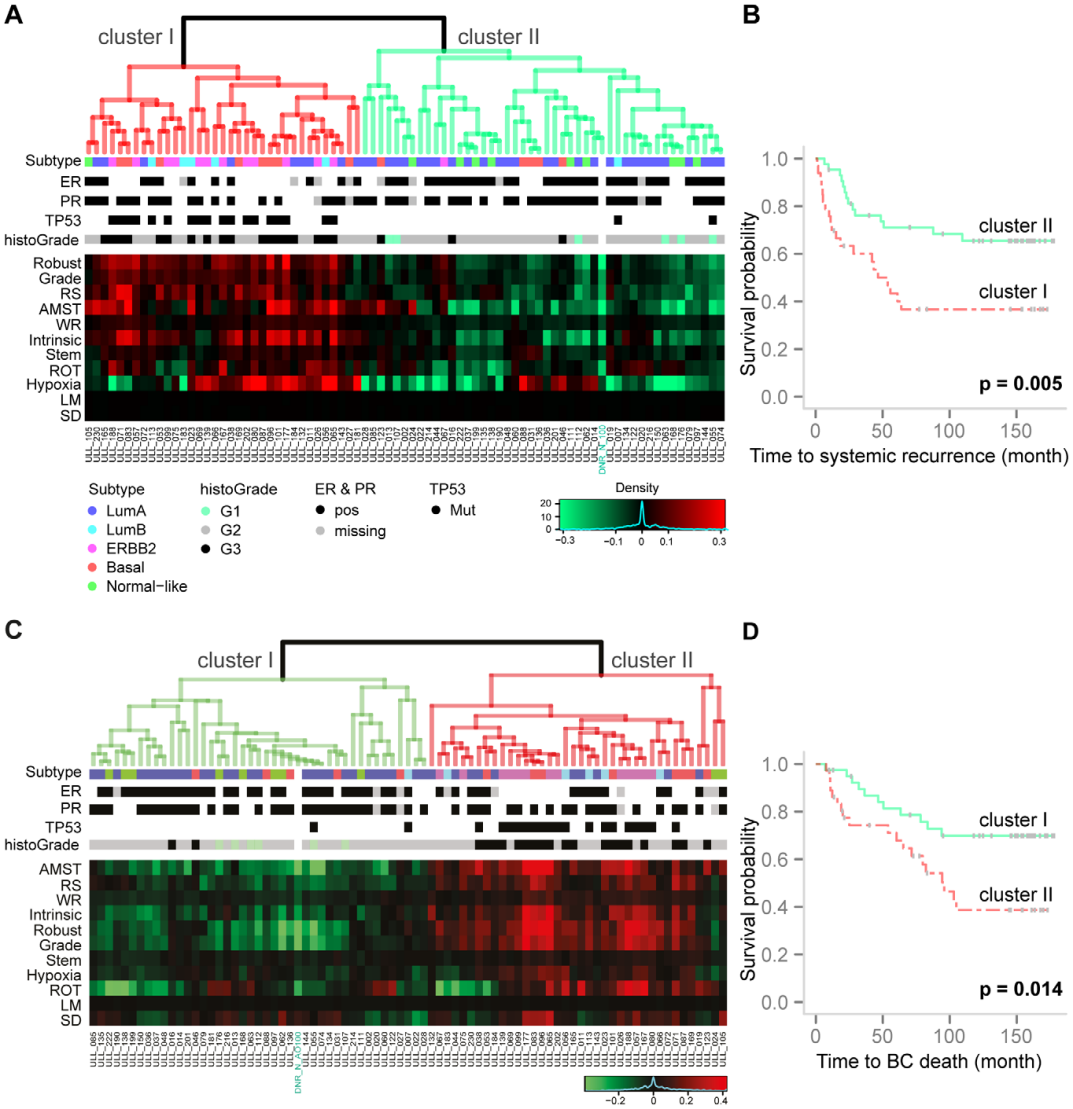
Hierarchical clustering of predicted PIs on test set and Kaplan-Meier analysis of the clusters. Results for systemic recurrence are in (A–B); for BC specific death in (C–D). In heatmaps (A, C), rows are notations for the gene sets. Columns are annotation for the patients; data outside of 1% quantile were trimmed. “Average” linkage based on Spearman correlation was used to construct the dendrograms. Figure A and C share legends for the clinical parameters. (**A**) Heatmap of predicted PIs on the test set for systemic recurrence from each gene sets. Two risk groups were observed from the hierarchical clustering; cluster I and cluster II. The control sample in Ull DNR_N_100, marked by green, was classified in the cluster associated with a lower risk (cluster II). (**B**) The Kaplan-Meier curves for the two clusters. A significant separation between the two groups was observed (χ^2^ = 7.8, df = 1, *p* = 0.005). (**C**) Heatmap of predicted PIs on the test set for BC specific death from each gene sets. Two risk groups were observed from the hierarchical clustering; cluster I and cluster II. The control sample in Ull DNR_N_100, marked by green, was classified in the cluster associated with a lower risk (cluster I). (**D**) A significant separation between the two Kaplan-Meier curves associated with the clusters was observed (χ^2^ = 5.996, df = 1, *p* = 0.014).

The predicted PIs for the test patients by each of the individual gene sets in our study were stacked into a PI matrix where rows indicate the identity of the gene set, columns indicate the identity of the patient in the test set and each cell contains the predicted PI risk score for relapse of a specific patient by a specific gene set (Figure 1B). Hierarchical clustering of the patients based on the PI matrix revealed two risk groups with distinct clinical characteristics (Figure 3A) and associated with significantly different survival probabilities (Figure 3B: Logrank test: χ^2^ = 7.8, df = 1, *p* = 0.005). This is in line with the findings from the results on the training set (Figure S2; see Methods S1 Part IV for calculation of the estimated PIs on the training set and Part V for a brief discussion on the results). A control sample included in the Ull set (DNR_N_100; marked in green in Figure 3A, see Materials and Methods) was correctly classified to the low risk group. Clinical and molecular characteristics for each of the two clusters are summarized in Table 5. For the 77 patients in the test set, the low risk group consisted of 44 patients; 14 of them had developed systemic recurrence within the follow-up time period. Furthermore, 29 out of 38 luminal A tumors (76%), 5 out of 13 basal-like tumors (39%), 33 out of 45 ER positive tumors (73%), 32 out 51 PR positive tumors (63%), 2 out of 20 *TP53* mutated tumors (10%) and all 6 Grade 1 tumors (100%) belonged to this group. The high risk group consisted of 33 patients of whom 20 experienced relapse within the follow-up time period with 53.9 month median survival time. Moreover, 9 out of 38 Luminal A tumors (24%); 8 out of 13 basal tumors (61%) and almost all *TP53* mutated tumors (18/20, 90%) belonged to the high risk group.

**Table 5:** Clinical and molecular characteristics of the two risk groups from hierarchical clustering of the test patients based on the predicted PI matrix.

	Low risk	High risk
Number of patients	44	33
Number of events (Recurr.)	14	20
Median survival (month)	-	53.9
LumA (%)	29/38 (76)	9/38 (24)
Basal (%)	5/13 (39)	8/13 (61)
ER+ (%)	33/45 (73)	12/45 (27)
PR+ (%)	32/51 (63)	19/51 (37)
*TP53* mutated (%)	2/20 (10)	18/20 (90)
Grade 1 (%)	6/6 (100)	0/6 (0)

### Prediction by individual gene sets

**Figure 4:**
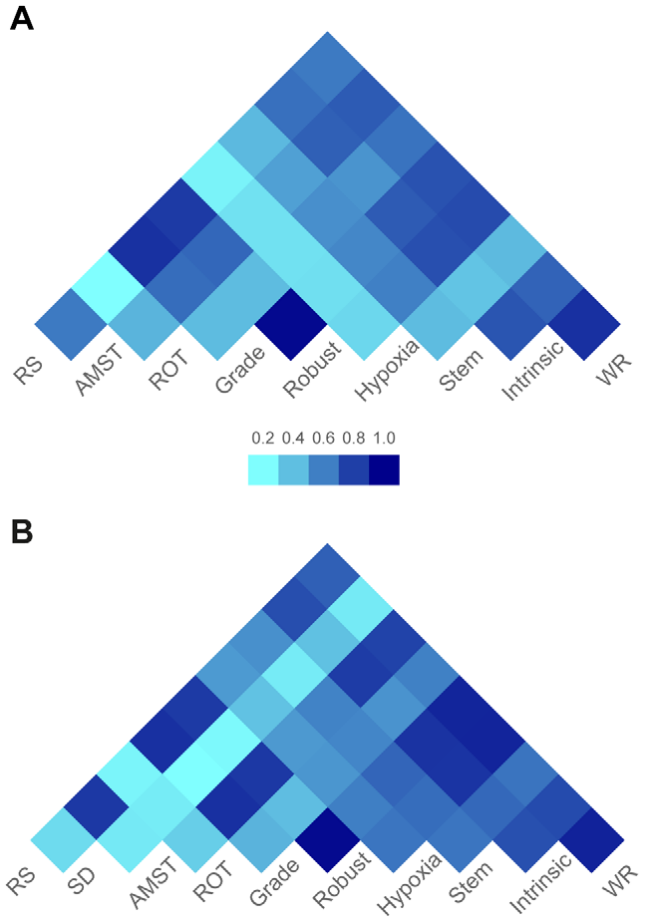
Correlation structure of predicted PIs from gene sets with convergence in model-building stage. Heatmap of Spearman correlation matrix of predicted PIs for corresponding survival endpoint from individual gene sets. (**A**) For systemic recurrence, nine gene sets that reached convergence during modeling building are displayed. (**B**) For BC specific death, ten gene sets that reached convergence during modeling building are displayed. Figure A and B share the same color legend.

The concordance structure for survival prediction among the studied gene sets is shown in the heatmap of the Spearman correlation matrix on continuous PI scales (Figure 4A). The gene sets *SD* and *LM* were left out since no optimal tuning parameter *λ* could be found. It should be noted that *Hypoxia* showed weak correlations with other gene sets, whereas *Robust*, *Grade* and *RS* were highly correlated.

**Figure 5:**
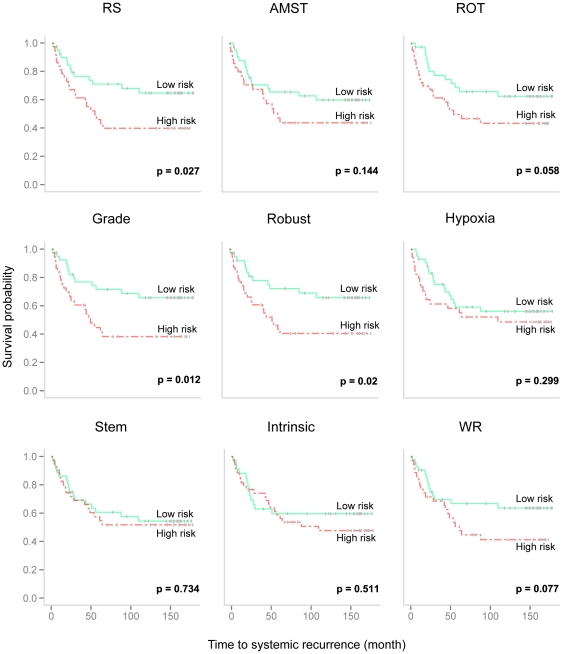
Systemic recurrence: Kaplan-Meier plot of the PI-risk groups for each of the individual gene sets. The Kaplan-Meier curves and the associated logrank *p* values for dichotomized PI-risk groups from each of the 9 converged gene-set models.

To increase the clinical applicability of PI scores, patients with a positive PI score were assigned to the high risk group and the remaining patients to the low risk group. The survival probabilities associated with the dichotomized risk groups were assessed by the logrank test. Kaplan-Meier plots for the predicted groups are shown in Figure 5 for each of the individual gene sets. Three gene sets were found to be significant: *Grade* (*p* = 0.012), *Robust* (*p* = 0.02) and *RS* (*p* = 0.027). Previously, we observed that predictions by these three gene sets were highly correlated, which explains their similar performances on the dichotomous scale of PI. In addition, two gene sets were borderline significant: *ROT* (*p* = 0.058) and *WR* (*p* = 0.077).

### Using principal components analysis to obtain a combined risk predictor

**Figure 6:**
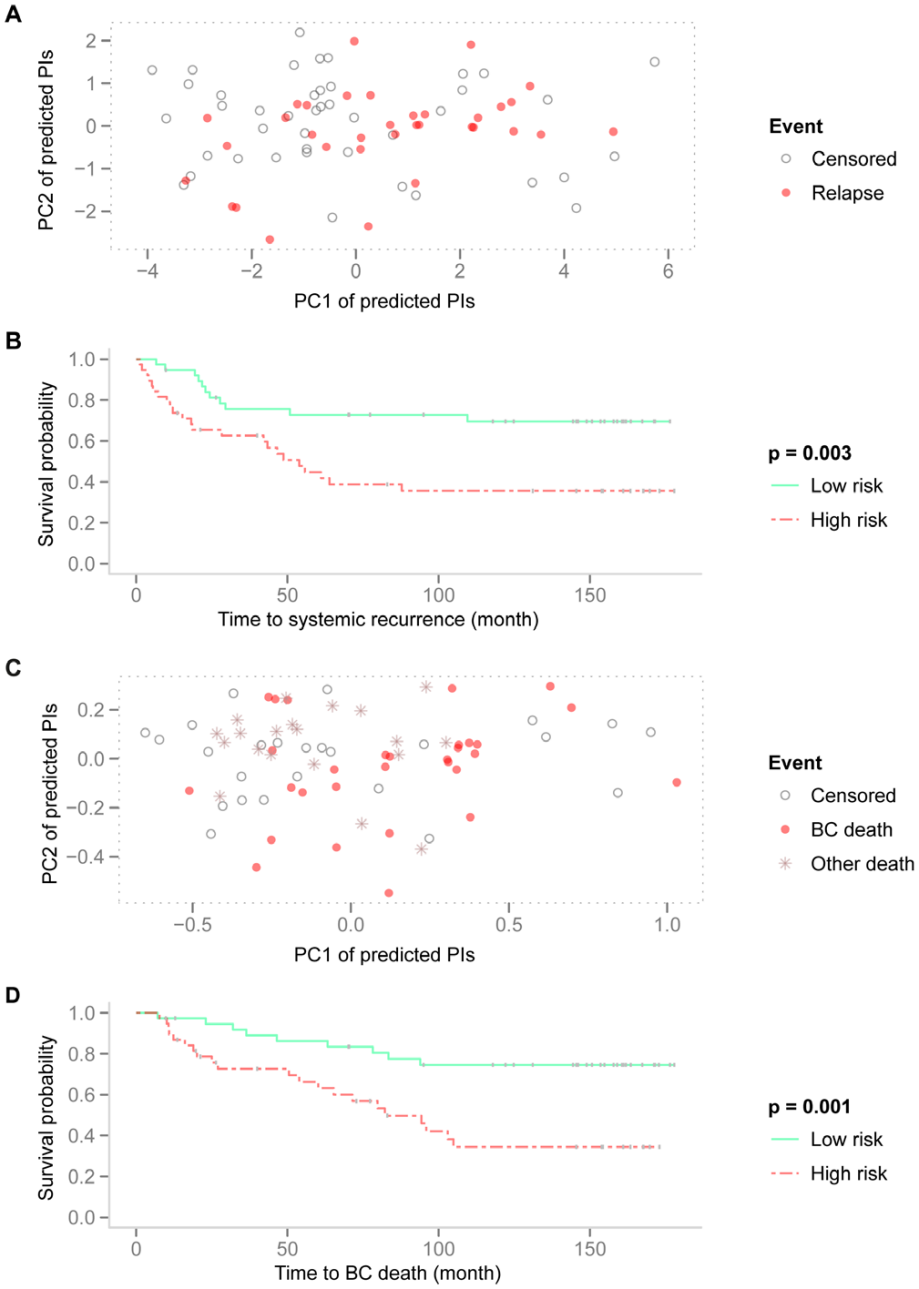
Systemic recurrence: PCA of predicted PIs from converged gene sets and performance of the resulting groups from PC1. Results for systemic recurrence are in (A-B); for BC specific death in (C-D). (A) Scatter plot of predicted PIs from 9 converged gene-set models on the space of the top two leading PCs. Black circles indicate censored observations; red dots indicate patients with relapse. (B) The Kaplan-Meier curves for high and low risk groups are significantly different (χ^2^ = 8.76, df = 1, *p* = 0.003). (C) Scatter plot of predicted PIs from 10 converged gene-set models on the space of the top two leading PCs. Black circles indicate censored observations; red dots indicate patients with BC specific death; brown stars indicate death from other reasons. (D) The Kaplan-Meier curves for high and low risk groups are significantly different (χ^2^ = 10.26, df = 1, *p* = 0.001).

The concordance structure among the studied gene sets for survival prediction (Figure 3A and Figure 4A) indicated that the main signal related to survival should be well captured by a lower dimensional representation of the PI vector associated with a patient. Consider the PI matrix (rows indicate gene sets and columns indicate patients) consisting of the predicted PIs of the test patients from the 9 converged gene sets in the model training. We used principal components analysis to derive linear combinations of the original nine-dimensional PI vectors that captured most of the variability of the PIs. Figure 6A shows a scatter plot of the combined risk prediction scores for the test patients on the first two principal components. The first principal component (PC1) captured 64% of the total variation and a total of 76% cumulative proportion of variation was captured by the first two components (Table S1). An increasing proportion of relapses were observed at the higher end of PC1. We created two classes by dividing the PC1 risk scores at the median value (−0.45); thus any patient with a PC1 risk score larger than −0.45 was considered to be a high-risk patient, and any patient with a PC1 risk score less than or equal to −0.45 was considered to be a low-risk patient. The two risk groups were associated with significantly different survival probabilities (Figure 6B, logrank test *p* = 0.003).

### Univariate comparison of predictors

**Figure 7:**
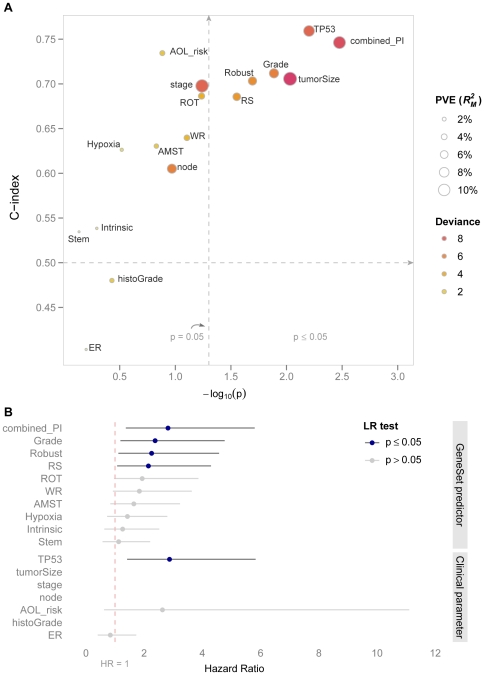
Univariate comparison of predictors for systemic recurrence. Comparison of combined-PI risk predictor with clinical parameters and individual gene-set predictors using univariate Cox model. (**A**) Y axis indicates C-index associated with individual predictor and X axis indicates the p values (on minus log10 scale) from likelihood ratio test in univariate Cox model. C-index  = 0.5 and the significant level: α = 0.05 for the likelihood ratio test are indicated by the dotted line. The size and the color of the bubble indicate the PVE and the deviance in univariate Cox model, respectively. The combined-PI risk predictor had the most significant marginal effect for predicting systemic recurrence (*p* = 0.003). It was associated with the second highest C-index score (C = 0.75) following *TP53* mutation status (C = 0.76). It had the second highest deviance (8.61) following tumor size (9.36), and the combined-PI predictor alone explained 10.6% of the variability as indicated by PVE, following tumor size (11.7%) and stage (11.1%) (**B**) X axis indicates HR from the univariate Cox model and the 95% CIs are shown along with the point estimates. “LR test” stands for likelihood ratio test. Insignificant predictors (likelihood ratio test *p*>0.05) are grayed out. To keep the results interpretable, only predictors with two levels are compared. The combined-PI risk predictor had the 2^nd^ largest HR (2.82 with 95% CI 1.37—5.80) following *TP53* mutation status (2.87 with 95% CI 1.42—5.83).

We performed univariate analysis for individual gene-set predictors as well as for clinical parameters including tumor size (pT1, pT2 and pT3-pT4), *TP53* mutation status, stage (1–4), node status (pN0, pN1, pN2-pN3 and pNx), ER status (positive versus negative), histological grade (1–3) and the Adjuvant! Online model (AOL), respectively. AOL is an established on-line breast cancer survival predictor; it calculates a 10-year survival probability based on the patient's age, tumor size, tumor grade, oestrogen-receptor status, and nodal status. Patients were assigned to the low risk group if their 10-year mortality risk was lower than 10% as predicted by Adjuvant! Online software. The dichotomized PI scores (positive scores indicate high risk and nonpositive scores indicate low risk) for the gene-set predictors were used in the univariate Cox model. The performance comparisons by using the likelihood ratio test, the deviance, the *proportion of variation explained* (PVE), the *concordance index* (C-index) and the *Hazard Ratio* (HR) are summarized in Table 6, Figure 7 and Table S2 for the combined-PI risk predictor (where we dichotomize on PC1) and other included predictors.

**Table 6:** PCA-combined PI risk predictor in univariate and multivariate Cox regression.

		Univariate				Multivariate			
*Covariate*		*p*	PVE*	Deviance	C§	HR [95% CI]	*p*	Partial PVE	C
Combined-PI risk	(overall effect)	0.003	0.106	8.611	0.746			11.3%	
	High (vs Low)					3.34 [1.49, 7.51]	0.004		
AOL risk		0.131	0.033	2.283	0.734				
*TP53*		0.006	0.092	7.47	0.759				
Tumor size	(overall effect)	0.009	0.117	9.358	0.706			5%	
	pT2 (vs pT1)					2.59 [0.86, 7.81]	0.091		
	pT3-pT4 (vs pT1)					2.97 [0.85, 10.42]	0.089		
Stage		0.058	0.111	7.501	0.698				
Node		0.107	0.076	6.090	0.605				
ER		0.634	0.003	0.227	0.403				
Histological grade		0.372	0.025	1.979	0.480				
RS		0.028	0.061	4.827	0.686				
AMST		0.148	0.027	2.092	0.630				
ROT		0.058	0.046	3.591	0.686				
Grade		0.013	0.077	6.174	0.712				
Robust		0.02	0.068	5.391	0.703				
Hypoxia		0.303	0.014	1.06	0.626				
Stem		0.734	0.001	0.115	0.535				
Intrinsic		0.508	0.006	0.437	0.538				
WR		0.079	0.039	3.093	0.640				
*Multivariate model*	Risk = Combined-PI + Tumor Size + strata(ER)						0.002	0.19 (R^2^)	0.705

For overall effect of the predictor in univariate Cox regression, Likelihood ratio test p value was reported. For individual levels within the predictor, Wald test *p* value was reported.

*PVE: proportion of variation explained in the outcome variable.

§ C: concordance index.

The combined-PI risk predictor was competitive in all the tested measurements. It showed the most significant effect on survival (*p* = 0.003) and it was associated with the second highest C-index score (C = 0.75) following *TP53* mutation status (C = 0.76). The information carried by the Deviance and PVE is highly consistent (Figure 7A). The combined-PI risk predictor had the second highest deviance (8.61) following tumor size (9.36), indicating good fit of the model. Furthermore, the predictor alone explained 10.6% of the variability as indicated by PVE, following tumor size (11.7%) and stage (11.1%); see Table 6. The high risk group assigned by the combined-PI risk predictor had a hazard rate of 2.82 (95% CI 1.37—5.80; Figure 7B; Table S2) times higher than the low risk group. Among all the tested predictors, *TP53* mutation status was the only factor that gave a slightly higher HR (2.87 with 95% CI 1.42—5.83; Figure 7B; Table S2).

The proportional hazards assumption, where the hazard ratios over time are constant, held in all the reported univariate Cox models, expect for ER status (*p* = 0.009; Table S2).

### Multivariate comparison of predictors

A multivariate Cox model was used to simultaneously assess the combined-PI risk predictor and the traditional clinical and molecular parameters that yielded significant results in the univariate comparison (*TP53* mutation status and tumor size). Due to the known association between ER status and survival, we included ER status as stratification variable and each stratum is permitted to have a different baseline hazard function while the coefficients of the remaining covariates are assumed to be constant across the strata. We observed a high correlation between *TP53* mutation status and the combined-PI risk predictor (odds ratio 15.0 (95% CI 3.1—145.7), Fisher's Exact test *p*<0.001). Analysis for model comparison showed that the combined-PI predictor added significant information to tumor size and *TP53* mutations (analysis of deviance *p* = 0.04; Akaike's Information Criterion (AIC) for model with and without combined-PI  = 191.8 and 194.01, respectively; Table S2). We used AIC in a hybrid stepwise strategy to build a final prognostic model where the combined-PI and tumor size were left as covariates and ER status as stratification variable in a Cox regression model (AIC: 191.46; Table S2. Likelihood ratio test *p* = 0.002; Table 6). The final model exhibited proportional hazards (*p* = 0.85; Table S2).

The combined-PI risk predictor had the most significant effect in the final multivariate model (*p* = 0.004). The HR for the combined-PI high-risk group was 3.34 (95% CI 1.49—7.51) compared with the low risk group. The partial PVE was calculated to indicate relative importance of the individual predictor in the multivariate setting: The combined-PI risk predictor captured 11.3% of the variability compared with 5% captured by tumor size. The C-index (C = 0.71) indicated satisfactory predictive discrimination ability for the final multivariate model.

### Breast cancer specific death as clinical endpoint

The proposed framework was also applied with breast cancer specific death as the clinical endpoint and the results are presented in Figure 2-4, 6. The predicted PI scores of the risk of dying from breast cancer by individual gene sets are summarized in the box plot in Figure 2B. The tuning parameters chosen by leave-one-out cross-validation are shown in Figure S3. The correlation structure indicates concordant predictions made by different gene sets (Figure 4B). Overall, the gene set SD had the weakest correlation to the other gene sets. Hierarchical clustering of the test set patients based on the predicted PI matrix defined to two risk clusters (Figure 3C) with distinct clinical characteristics and associated with significantly different survival probabilities (*p* = 0.014); see Figure 3D. Risk scores were combined as previously described into one risk score by projecting the PIs onto the first principal component (Figure 6C), in which 73% of the total variance was captured. The continuous score was further dichotomized into high risk and low risk using the median of the PC1 score. The difference in the associated survival probabilities of the two predicted risk groups was confirmed by the logrank test (*p* = 0.001; Kaplan-Meier plot in Figure 6D). For comparison, the univariate performances of individual gene sets for dichotomous prediction and clinical parameters as well as AOL are shown in Figure S4 and Methods S1. The combined-PI risk predictor for breast cancer specific death was the most significant among all the tested predictors in the univariate setting (likelihood ratio test *p* = 0.001) with the second largest HR 3.36 (95% CI 1.5—7.4), following *TP53* mutation status (HR 3.46 with 95% CI 1.7—7.2; Figure S4B). Furthermore, it had the second largest C-index (C = 0.77) following *TP53* (C = 0.8; Figure S4A) and it was also highly ranked by PVE (12.4%) and deviance (10.2) following node status (PVE = 12.5%, Deviance = 10.3; Figure S4A). The combined-PI risk predictor was the most significant predictor (*p* = 0.005; Methods S1) with HR 4.62 (95% CI 1.58—13.55) in a multivariate Cox model containing combined-PI risk, *TP53* mutation status (HR = 2.6, 95% CI 0.86—7.78), tumor size and node status, stratified by ER status (See Methods S1 for the AIC stepwise model selection). The correlation between the combined-PI predictor and *TP53* mutation status appeared to be significant (odds ratio 33.9 with 95% CI 4.8—1490.4, Fisher's Exact test *p*<0.001; Methods S1) and the combined-PI added significant information to *TP53*, tumor size and node status (analysis of deviance *p* = 0.035; AIC for model with and without combined-PI  = 149.96 and 152.41, respectively; Methods S1). Except for the univariate Cox model for ER status, the proportional hazards assumption was met for the reported models (Methods S1).

### Assess robustness of the combined-PI model

To test the robustness of the proposed procedure, we performed the analyses by switching the test and training sets. To gain computational efficiency, we applied 10-fold CV instead of LOOCV during the model training procedure for each of the gene sets. We obtained similar results for prediction of BC specific death (Figure S5E–H). The combined-PI predictor and *TP53* mutation status was strongly correlated (odds ratio 11.2 with 95% CI 4.0—36.8, Fisher's Exact test *p*<0.001). For prediction of systemic recurrence, only three out of the eleven tested gene sets achieved model convergence, which most likely contributed to a compromised combined-PI risk predictor built from insufficient signals (Figure S5A–D).

Furthermore, we also checked the stability of the combined-PI prediction by correlating the predicted combined-PIs on the test set from one analysis with the combined-PIs on the training set from the switched analysis. The continuous PCA combined-PI scores were used instead of dichotomizing on PC1. We performed this screening for the prediction of BC specific death, and observed moderate correlations of the combined-PIs on the MicMa set (0.48) as well as on the Ull set (0.63). This indicates clearly that there is a fair correlation between the combined-PIs when trained on different data sets, but also that there is substantial variation.

## Discussion

High-throughput genome profiling for genetic marker discovery is widely applied in the field of genomic and personalized medicine. For example, a gene signature characterizes certain biological aspects and/or may be used to predict disease outcome by mathematically combining the expression values of a set of genes. The methods generating the combined expression pattern of the biomarkers differ from study to study. A gene signature therefore consists of a group of gene identities together with a distinct classifier or a predictor to predict disease outcome.

In the present work, we applied Cox regression to reconstruct a classifier from the same set of original genes as a number of published gene signatures. Only the associated gene identities from the original signature are retained, while the classifiers themselves are derived on the basis of the same modeling procedure and the same tumor data set. As a consequence, the resulting gene signatures are derived on the basis of the same clinical endpoint (systemic relapse or breast cancer specific death). The gene sets in our study were pre-selected based on prior knowledge in breast cancer. We are interested in survival prediction by the collective expression pattern of the whole gene set without additional gene selection. Furthermore, since the number of features (genes) outnumbers the number of samples in the training set, p *> N*, for most of the gene sets of interest, direct estimation of the coefficients using standard Cox regression is unfeasible. Even for those gene sets with p *< N,* we still have p large enough to render the coefficient estimates highly unstable and thus of doubtful value for prediction. Accordingly, we perform parameter estimation using a penalized partial likelihood criterion [17] that forces the solution to the estimation problem to have small L2-norm. Using an L2 penalty trades a little bias for a larger reduction in variance to reduce the prediction error on a new data set. This is the so-called “bias and variance tradeoff” [18]. Cox-ridge regression has been shown to be an effective model in survival prediction using gene expression data [19], [20]. There is no actual variable selection involved; while genes are generally down weighted, all the genes included in the Cox model will be present in the final model.

We observed that two of the pre-selected gene sets failed for survival prediction at the model training stage: gene set LM for both tested clinical endpoints, and gene set SD for systemic relapse. The reason for their poor performance is likely related to the lack of comparability of the data and methods used to construct the original gene signatures and those used to construct ours. Gene set LM contains 54 genes that mediate risk of breast cancer metastasis to lung [7]. The original risk index for lung metastasis was defined as a linear combination of gene expression values weighted by their estimated Cox regression parameters. The essential biological information captured in the original Minn et. al dataset [7] was absent from the cohorts studied in the present work, which contain mainly early-stage breast cancers. Given the comparable methods in classifier construction, we believe the biological incomparability of data sources used for model building led to the poor performance of gene set LM in our study. Likewise, the gene set SD might not have been expected to perform well due to the intrinsic biological differences in the training sets used for the present study and the original study, where microdissected stroma from breast cancer specimens were used to identified these 26 stroma-derived prognostic genes [21]. Not all gene sets that were left for the model evaluation achieved significant survival prediction in the studied test set (Figure 5 and Table 6). The inter-cohort heterogeneity most likely played a role. The studied training and test set showed borderline significantly different survival probabilities for the survival endpoints (systemic relapse *p* = 0.0765; breast-cancer specific death *p = *0.0564; Figure S6). Unless there exists a “gold-standard” dataset representing perfectly the underlying population for breast cancer, dealing with the heterogeneity feature is inevitable. The proposed framework demonstrates a straightforward yet effective approach to improve the survival prediction power by integrating multiple gene set predictors.

Interestingly the combined-PI risk predictor correlates with but provides additional information to *TP53* mutation status. *TP53* mutation status has been shown to be one of the strongest single molecular prognostic markers in breast cancer. It is known as a key molecule involved in different pathways important in cancer. It is a molecular marker important to compare with other prognostic markers to gain insights about the underlying biology. It is a prime example of a single molecule not function alone, but rather involving many players in various networks. Restoring *TP53* activity is a potential therapeutic strategy [22].

The results indicated that to some extent the generalization from MicMa set to Ull set was better than that from Ull to MicMa. We suspect the main reason may be the reduction of the training set sample size from 123 in the MicMa cohort to 80 in the Ull cohort. Since the training step is prone to over-fitting due to the large number of genes from which the models may be fitted, the ability to fit a reliable model strongly depends on having a sufficient sample size.

As a potential extension of the framework of mining large collection of gene sets for survival prediction, an optional filter step could be added prior to the analysis to eliminate gene sets that are not significantly related to survival or not enriched in the training set. Our analysis framework does not restrain to the gene sets from gene signatures, as this is one of the many ways to provide input to the “combining power procedure”.

In conclusion, our proposed framework for improving survival prediction contains three analytical modules: (1) gene signature (gene-set prognostic model) construction, (2) dimension reduction and (3) risk prediction. Each module could be fine tuned or modified depending on the data under study. Our study showed that by aggregating the predictive strength from multiple gene sets we can improve the outcome prediction in breast cancer, and it can be broadly applicable to breast cancer survival risk assessment.

## Materials and Methods

### Ethics statement

The studies included in the project were approved by the Regional Ethical Committee (REK: S97103 for MicMa and REK: 200401129-1 for Ull). All samples were obtained with written informed consent approved by the ethical committee.

### Tumor samples and patients

The training set (MicMa) comprise published gene expression and clinical data on 123 human breast cancer cases, mainly stage I and II [11]. Patients were treated for localized breast cancer and included between 1995 to 1998 [23]. The prognostic model for relapse was trained using 118 patients with available endpoint status. All the 123 patients were available for training the prognostic model for breast cancer specific death. The test set (Ull) mainly consisted of early stage breast cancers from which gene expression and clinical data were available [16]. Patient samples were sequentially collected from 1990 to 1994. In this data set, together with eighty tumor cases a normal breast tissue sample coded as “DNR_N_AO100” was included as control. Descriptive aspects of the training and test sets are given in Table S3. More details are provided in Methods S1.

### Microarray

Both the training set (MicMa) and test set (Ull) were obtained using the Stanford 43k cDNA microarrays. Using the protocols described in Methods S1, total RNA was isolated, amplified, labeled and hybridized to arrays containing around 42,000 features representing 23169 unique cluster IDs (UniGene Build Number 215) produced at the Stanford Functional Genomics Facility (http://www.microarray.org/sfgf/jsp/home.jsp). All procedures are available at Stanford Genomics Breast Cancer Consortium Portal (http://genome-www.stanford.edu/breast_cancer/) for the MicMa set and at the referred web site (http://www.stanford.edu/group/sjeffreylab/) for the Ull set. All data is MIAME compliant. Raw data for MicMa set has been deposited in the NCBI's Gene Expression Omnibus database (GEO; http://www.ncbi.nlm.nih.gov/geo/) with accession number GSE3985. Both datasets are accessible through Stanford Microarray Database (SMD; for the MicMa set: http://smd.stanford.edu/cgi-bin/publication/viewPublication.pl?pub_no=833; for the Ull set: http://smd.stanford.edu/cgi-bin/publication/viewPublication.pl?pub_no=629).

### Preprocessing

For the MicMa set, normalized (loess with print tip stratification) log2-transformed gene expression ratios were retrieved from SMD (http://smd.stanford.edu/), filtered for spot intensity over background at least 1.5 in both sample and reference, and finally, filtered for those genes that fulfilled the spot filter criteria in at least 85% of the experiments. For the Ull set, probes were filtered for spot quality and included in the analysis if the pixels within a spot showed a regression correlation of at least 0.6 or if the signal intensity of both sample and reference were at least 1.5 over background; data were further normalized by the default total Intensity Normalization in SMD (http://smd.stanford.edu/help/results_normalization.shtml). For both MicMa and Ull, k-nearest neighbor (KNN) imputation [24] with k = 10 was applied to impute missing values in the filtered expression dataset.

### Gene sets

An overview of the 11 published gene sets studied in this report are listed in Table 1. The abbreviated coding for the individual gene sets are used instead of the full name throughout this paper. The annotation files for the gene sets were downloaded from the web sites indicated in the original publications or were requested directly from the authors. Brief discussions of various gene sets are presented in the Methods S1.

### Adjuvant! Online model

Adjuvant! [25] (https://www.adjuvantonline.com) uses patient age, comorbidity level, ER status, tumor grade, tumor size and number of positive lymph nodes to predict 10-year risk for breast cancer mortality. It also predicts the benefit of adjuvant therapy for women with early-stage breast cancer. A description of the Adjuvant! Online model and the details for computing risk scores of the test patients can be found in Methods S1. Low risk is defined in this paper as lower than 10% 10-year risk of breast cancer mortality. A total of 72 patients in the Ull data set with suitable characteristics for Adjuvant! Model were entered into the comparison study.

### Molecular subtype assignment

Tumors were assigned to a subtype using Pearson correlation to the expression centroids as previously described [16], [26].

### Cross-platform gene identity mapping

Most of the gene sets in the study were developed from microarray platforms different from Stanford 43k cDNA array (see Table 1). For gene sets developed from Stanford 43k cDNA arrays, clone IDs were used as the “linker” to link the gene identifiers in the gene set to cDNA. For gene sets developed from different platforms, gene identifiers in the gene set were mapped to the cDNA clones using the following linkers: UniGene, gene symbol and gene alias, where UniGene had the highest priority while gene alias had the lowest priority. When matches occurred by using multiple linkers, we took the matches from the highest ranked linker. Furthermore, we collapsed the matched clones representing the same gene by their mean expression value (Figure 1A).

Annotations for Stanford 43k cDNA array were retrieved from SMD SOURCE (http://smd.stanford.edu/cgi-bin/source/sourceSearch) under UniGene Build Number 215. And annotations for the individual gene sets were retrieved either from manufacture chip annotation files or from SMD.

### Penalized Cox regression for survival prediction based on individual gene sets

We assume that at any given time, a breast cancer patient has a certain risk of experiencing a specific event, which in our case is either relapse after primary surgery or breast cancer related death. We furthermore assume that for a number of patients we have measured the time to this event. Patients that experience none of these events for the duration of the study are labeled as censored. For such patients, the recorded time is simply the last point of observation and carries a different interpretation than a time to an event. To associate the risk of an event to observed features, we consider a Cox's proportional hazards model (*Cox model*) with the expression of selected genes as the covariates. Suppose that for a patient we have observed a total of *p* expression values 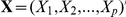. In principle, we may model the risk of relapse/death as a function of all measured expression values, as in the Cox model 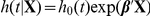, where the interpretation is that the instantaneous risk (also known as the *hazard*) of experiencing the event at time *t* is a product of two functions, the first depending only on the time point (and not on the particular patient at hand), and the second depending only on the expression values of the patient (and not on the time point). In a Cox model, the relative hazard between two individuals with expression vectors 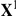 and 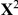
respectively is expressed by the quantity
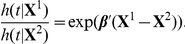



Accordingly, even though the hazard fluctuates over time for any one individual, the relative hazard between individuals is constant over time. Let 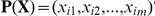 denote a subselection of genes (variables) from the complete list of *p* genes. Considering the risk of relapse/death as a function of the selected genes only, we obtain the Cox model 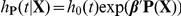. In this paper, we want to model risk of relapse/death as a function of R = 11 gene sets of individual sizes 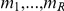, each corresponding to a particular subselection of genes. Thus, we consider the Cox models
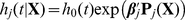
(1)where *j = 1,2,…,R* indexes the gene sets, 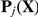 is the selected genes (variables) in the *j*th gene set, 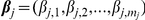 is the vector of coefficients (weights) associated with the genes in the *j*th gene set, and 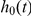 is the (common) baseline hazard function. Since the response of interest is a possibly censored survival time, for a given gene set, a Cox proportional hazards model was used to describe the risk of a patient experiencing an event in response to expression of gene covariates. The expression values for each gene were mean centered and scaled to unit standard deviation in both the training and the test datasets. All Cox models in the gene signature construction part of the analysis were fitted using an L2-penalized partial Cox likelihood, whereas the Cox model in the multivariate comparison was fitted using the ordinary partial Cox likelihood. To determine the penalty parameter, we applied the leave-one-out cross validation procedure proposed by Verweij and van Houwelingen [17] (See Methods S1).

A total of 118 MicMa patients with available information on systemic recurrence status were used to develop a prediction model using Cox-ridge regression for a gene set. For the *j*'th gene set (*j* = 1, …, 11), the optimal gene-set specific tuning parameter 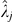
was found by the leave-one-out cross validation procedure [17] using the training set; we then estimated the coefficient vector 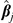 associated with individual genes in gene set *j* by the Cox-ridge model (1) (Figure 1A). The predicted prognostic index for the *j*th gene set and the *i*th patient in the test data set was calculated as 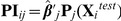 which is sum of weighted gene expression of the test patient *i* (Figure 1A). The weights 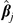 for individual genes in the *j*th gene set were the corresponding estimated coefficients from the training set using the Cox-ridge model (1) (Figure 1A). See Methods S1 for details.

### Prediction performance evaluation for individual gene set

Spearman correlations were used to show the concordance structure for risk prediction among the gene sets. The change in deviance (ΔD) is an indicator of predictive performance of a model obtained from a training dataset on a novel test dataset and is given by 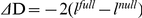 where 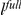 is the optimal log-likelihood with all the genes in the gene set included, and 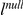 is the optimal log-likelihood with no genes included. The continuous PI scores were dichotomized into high- and low-risk groups using zero as cutoff, where a positive PI score indicates high risk. The resulting PI risk groups for individual gene set were tested in a univariate Cox model and the difference of the Kaplan-Meier survival curves of the groups were also tested by logrank test.

### Hierarchical clustering

As a graphical illustration of the relationship among the gene sets, the predicted PIs for all patients in the test set (Ull) using each of the gene sets were clustered using hierarchical clustering with Spearman correlation and average distance. The Kaplan-Meier survival curves were plotted for the resulting groups and the differences in clinical indications among the clusters were tested by a logrank test.

### Dimension reduction by principal components analysis

Principal components analysis (PCA) was used to project the PIs from each of the individual gene sets onto a lower dimensional space (Figure 1B). The values off the first principal component were used as the combined risk scores, and further dichotomized by median cut, where a patient with a score on PC1 higher than median values on PC1 was considered to be high risk. A logrank test was carried out to assess the significance of the differences in survival probabilities associated with resulting dichotomous risk groups (Figure 1B). The method of choice was PCA based on the covariance matrix since we expected PIs with higher variance to carry more information than PIs with less variance. In analyses of relapse, however, this ended up dominated by Hypoxia although this correlated little with other PIs, and was therefore replaced by PCA based on the correlation matrix.

### Univariate comparison of predictors

Univariate Cox models were used to compare the effects of the *combined-PI risk predictor,* individual gene signatures (dichotomized by splitting at 0), clinical parameters (Tumor size and Histological grade, *TP53*, Node status, ER status and Stage), as well as the Adjuvant! Online model. A *likelihood ratio test* was used to assess the significance of the overall effect of a predictor in the univariate models. In addition, *deviance* was used to check the goodness of the model fit. The marginal contribution by a single predictor in the univariate setting was evaluated using the *proportion of variation explained* in the outcome variable (PVE) [27]. PVE 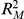, comparable with the R^2^ in regression modeling is an indicator that quantifies the importance of covariates in the Cox model.

The *Hazard Ratio* (HR) was used as an accuracy measure for the risk group prediction for different predictors. In the univariate setting, HR is a summary of the risk difference between patient groups defined by the predictor. To keep the results interpretable and comparable, we presented the HRs for the predictors with two risk groups (excluded Tumor size, Histological grade, Node status and Stage). The larger the HR, the better is the discrimination between the groups of the patients, such as low- and high-risk.

The *concordance index* (C-index) [28], an analogy to area under the receiver operating characteristic (ROC) curve in survival analysis, was computed to assess the predictive discrimination ability of each of the predictors in the corresponding univariate Cox model (Methods S1). It measures the probability of concordance between the predicted and observed responses in terms of lengths of time to event of any two patients. The larger the C-index, the better is the predictability of a survival model. A value of 0.5 indicates no predictive discrimination and a value of 1 indicates perfect separation of patients with different outcomes [28].

### Multivariate comparison of predictors

The significant predictors in the univariate analysis were included in a multivariate Cox model. Model selection was carried out using Akaike's Information Criterion (AIC) [29] and analysis of deviance. The relative importance of a covariate in a multivariate Cox model was measured by the partial PVE, which was calculated as the difference between 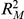 for the full model and 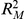 for a model with a factor of interest excluded. See Methods S1 for details.

### Software

All analyses were performed in R (version 2.11.1), which is available at http://cran.r-project.org/. The R package “penalized” [30] was to perform penalized Cox regression. R code for the procedures described in this paper are available from the correspondence author.

## Supporting Information

Figure S1 **Systemic recurrence: Cross-validated likelihood profile on** ***λ*** **grid.** The dotted line indicates the location of the optimal *λ* value *λ*opt for each gene set. RS: 338; SD: Inf; LM: Inf; AMST: 227; ROT: 1029; Grade: 3580; Robust: 1705; Hypoxia: 392; Stem: 3623; Intrinsic: 1576; WR: 11261. Modeling gene set SD and LM did not reach convergence by the specified criteria in the study.
(TIF)

Figure S2 **Systemic recurrence: Hierarchical clustering of estimated PIs for systemic recurrence on training set and the resulting risk clusters in a Kaplan-Meier plot.** (**A**) Heatmap of estimated PIs on training set for systemic recurrence from each gene sets. Rows are notations for the gene sets. Columns are annotation for the patients; data outside of 1% quantile were trimmed. “Average” linkage based on Spearman correlation was used to construct the dendrograms. Two risk clusters I and II, were observed from the hierarchical clustering with distinct clinical characteristics: a total of 30 out of 49 Luminal A tumors (61%) were clustered in the low risk group, and 19 luminal A tumors (39%) were found in the high risk group. (**B**) The Kaplan-Meier curves for cluster I and cluster II. A significant separation between the two clusters was observed (χ2 = 49.7, df = 1, *p*<0.001).
(TIF)

Figure S3 **BC specific death: Cross-validated likelihood profile on** ***λ*** **grid.** The purple dotted line indicates the location of the optimal *λ* value *λ*opt for each gene set. RS: 343; SD: 249; LM: Inf; AMST: 230; ROT: 379; Grade: 1337; Robust: 898; Hypoxia: 1823; Stem: 3866; Intrinsic: 1639; WR: 8317. Modeling gene set LM did not reach convergence by the specified criteria in the study.
(TIF)

Figure S4 **Univariate comparison of predictors for BC specific death.** (**A**) Y axis indicates C-index associated with individual predictor and X axis indicates the p values (on minus log10 scale) from likelihood ratio test in univariate Cox model. C-index  =  0.5 and the significant level: α  =  0.05 for the likelihood ratio test are indicated by the dotted line. The size and color of the bubble indicates the PVE and deviance in univariate Cox model, respectively. The combined-PI risk predictor for BC specific death was the most significant one among all the tested predictors (likelihood ratio test *p*  =  0.001). It had the second largest C-index (C  =  0.77) following *TP53* (C  =  0.8). And it was also highly ranked by PVE (12.4%) and deviance (10.2) following node status (PVE  =  12.5%, Deviance  =  10.3). (**B**) X axis indicates HR from the univariate Cox model and the 95% CIs are shown along with the point estimates. “LR test” stands for likelihood ratio test. Insignificant predictors (likelihood ratio test *p* > 0.05) are grayed out. The combined-PI risk predictor had the second largest HR 3.36 (95% CI 1.5—7.4), following *TP53* mutation status (HR 3.46 with 95% CI 1.7—7.2).
(TIF)

Figure S5 **Summary of switched analysis by using Ull as training set and MicMa as test set.** Results for systemic recurrence are in (A-D); for BC specific death in (E-H). (**A**) Boxplot of predicted PI for systemic recurrence. Only three out of eleven gene sets had converged model in model training stage. (**B**) Spearman correlation structure among gene sets with convergence. (**C**) Projection of the predicted PIs on space formed by PC1 (captured 81% variability) and PC2. (**D**) Kaplan-Meier curves associated with the two risk groups by median-cut of PC1 value (logrank *p*  =  0.191). (**E**) Boxplot of predicted PI for BC specific death. (**F**) Spearman correlation structure among gene sets with convergence. (**G**) Projection of the predicted PIs on space formed by PC1 (captured 65% variability) and PC2. (**H**) Kaplan-Meier curves associated with the two risk groups by median-cut of PC1 value (logrank *p*  =  0.028).
(TIF)

Figure S6 **Survival curves for training set (MicMa) and test set (Ull).** The logrank test showed that the training cohort and test cohort had borderline significant survival curves for the survival endpoint.
(TIF)

Table S1 **Results summary of PCA on predicted PIs for systemic recurrence from 9 converged gene sets.**
(PDF)

Table S2 **Results summary of univariate and multivariate analysis of combined PI-risk predictor for systemic recurrence.**
(PDF)

Table S3 **Molecular and clinicopathological characteristics of the tumor material in MicMa and Ull datasets.**
(PDF)

Methods S1 **Supplementary methods, results, notes, and references.**
(PDF)
