# Supplementary figures and images for: Combining Gene Signatures Improves Prediction of Breast Cancer Survival
Source: PLoS One. 2011 Mar 10;6(3):e17845. doi: 10.1371/journal.pone.0017845 (PMC3053398; doi:10.1371/journal.pone.0017845)

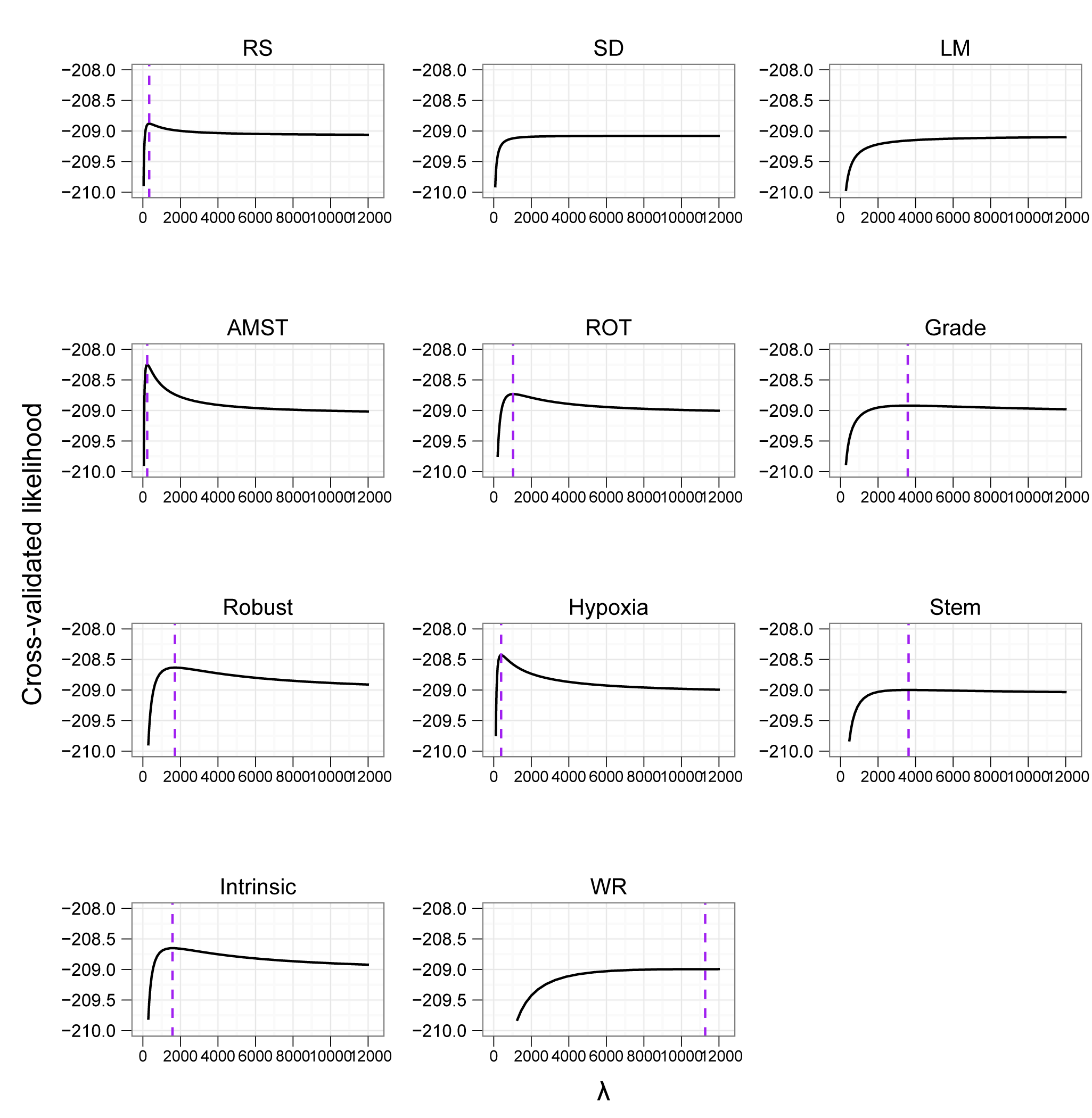

Supplement: Figure S1 — Systemic recurrence: Cross-validated likelihood profile on λ grid. The dotted line indicates the location of the optimal λ value λopt for each gene set. RS: 338; SD: Inf; LM: Inf; AMST: 227; ROT: 1029; Grade: 3580; Robust: 1705; Hypoxia: 392; Stem: 3623; Intrinsic: 1576; WR: 11261. Modeling gene set SD and LM did not reach convergence by the specified criteria in the study. (TIF) [file pone.0017845.s001.tif]

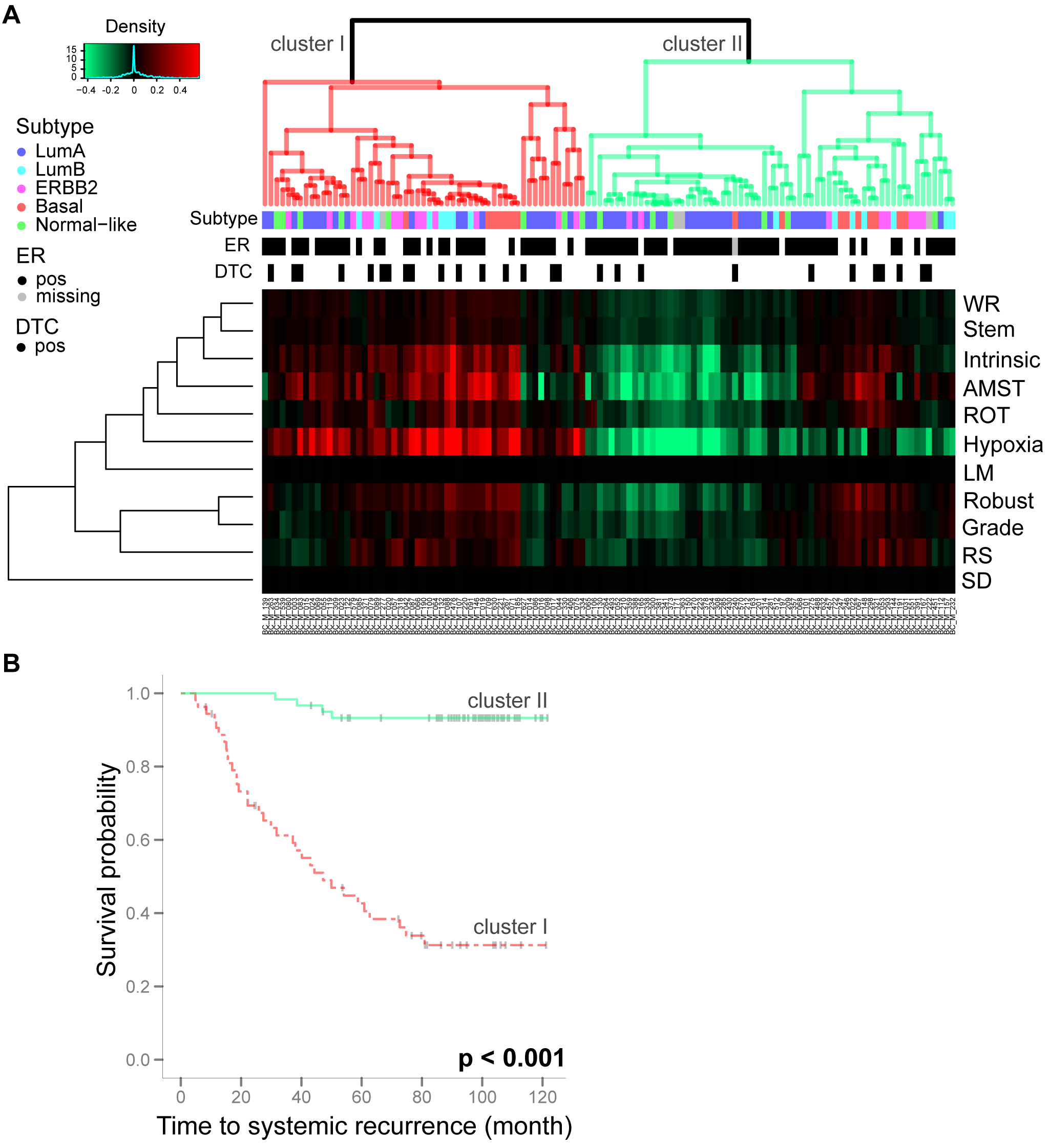

Supplement: Figure S2 — Systemic recurrence: Hierarchical clustering of estimated PIs for systemic recurrence on training set and the resulting risk clusters in a Kaplan-Meier plot. (A) Heatmap of estimated PIs on training set for systemic recurrence from each gene sets. Rows are notations for the gene sets. Columns are annotation for the patients; data outside of 1% quantile were trimmed. “Average” linkage based on Spearman correlation was used to construct the dendrograms. Two risk clusters I and II, were observed from the hierarchical clustering with distinct clinical characteristics: a total of 30 out of 49 Luminal A tumors (61%) were clustered in the low risk group, and 19 luminal A tumors (39%) were found in the high risk group. (B) The Kaplan-Meier curves for cluster I and cluster II. A significant separation between the two clusters was observed (χ2 = 49.7, df = 1, p<0.001). (TIF) [file pone.0017845.s002.tif]

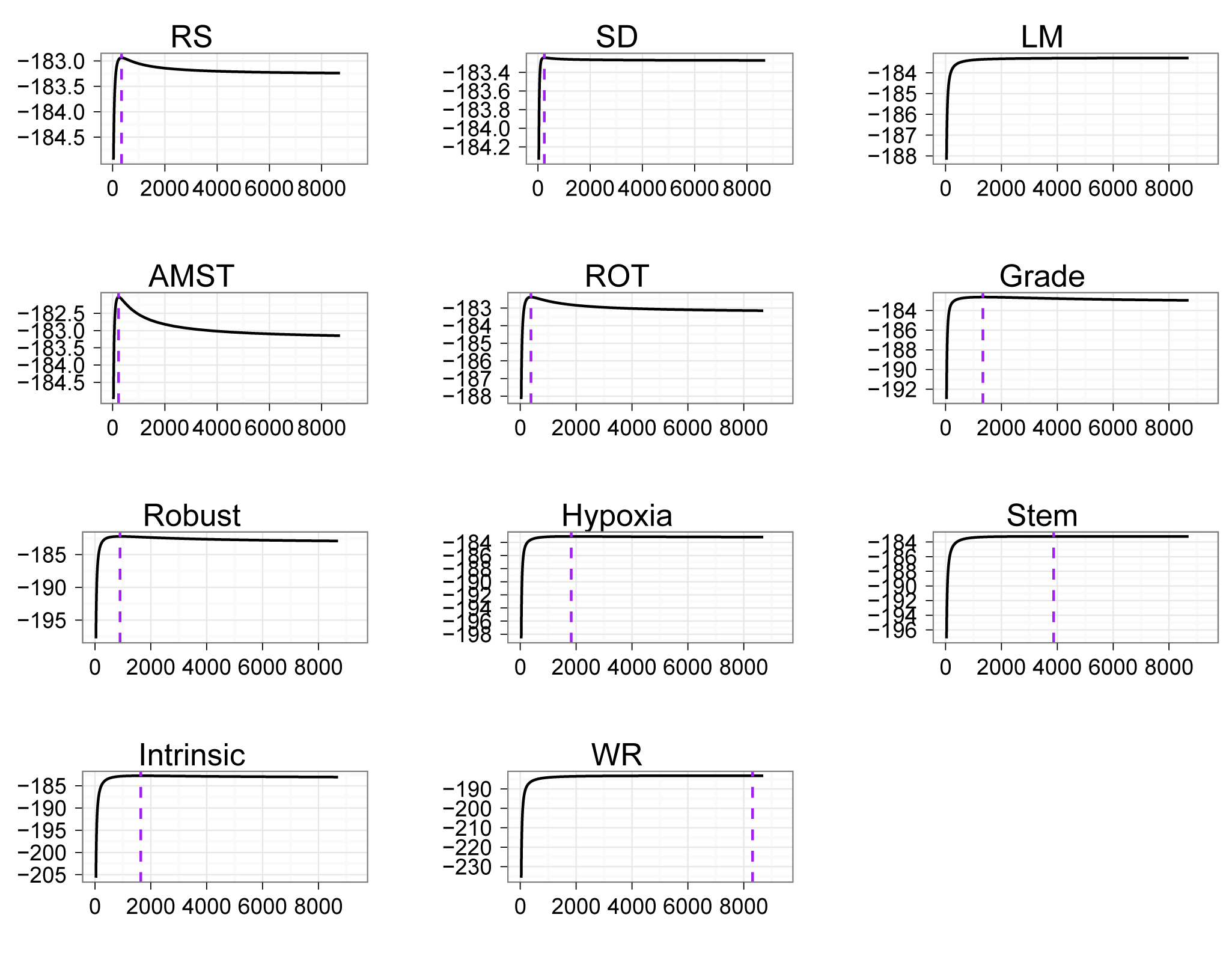

Supplement: Figure S3 — BC specific death: Cross-validated likelihood profile on λ grid. The purple dotted line indicates the location of the optimal λ value λopt for each gene set. RS: 343; SD: 249; LM: Inf; AMST: 230; ROT: 379; Grade: 1337; Robust: 898; Hypoxia: 1823; Stem: 3866; Intrinsic: 1639; WR: 8317. Modeling gene set LM did not reach convergence by the specified criteria in the study. (TIF) [file pone.0017845.s003.tif]

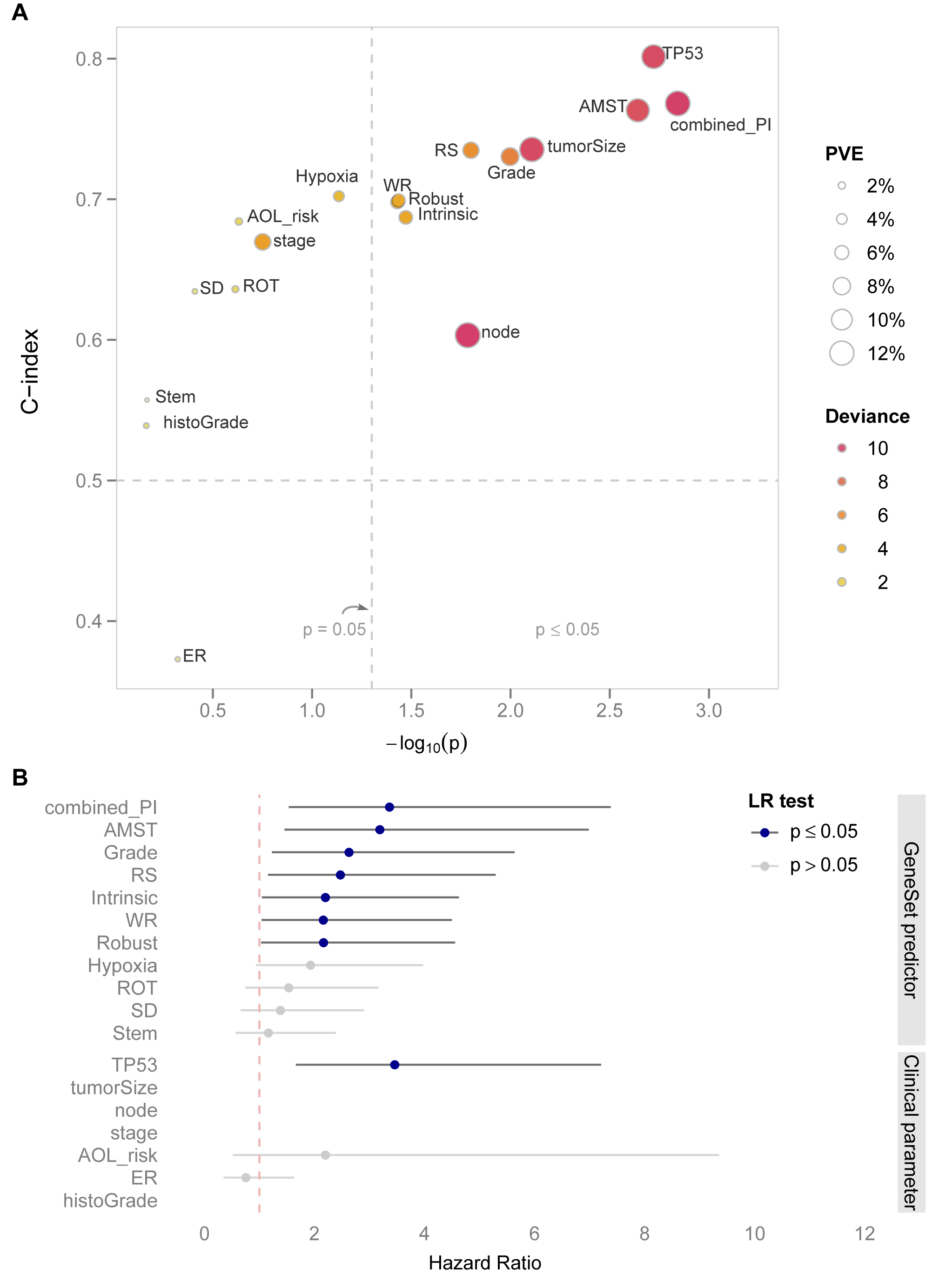

Supplement: Figure S4 — Univariate comparison of predictors for BC specific death. (A) Y axis indicates C-index associated with individual predictor and X axis indicates the p values (on minus log10 scale) from likelihood ratio test in univariate Cox model. C-index = 0.5 and the significant level: α = 0.05 for the likelihood ratio test are indicated by the dotted line. The size and color of the bubble indicates the PVE and deviance in univariate Cox model, respectively. The combined-PI risk predictor for BC specific death was the most significant one among all the tested predictors (likelihood ratio test p = 0.001). It had the second largest C-index (C = 0.77) following TP53 (C = 0.8). And it was also highly ranked by PVE (12.4%) and deviance (10.2) following node status (PVE = 12.5%, Deviance = 10.3). (B) X axis indicates HR from the univariate Cox model and the 95% CIs are shown along with the point estimates. “LR test” stands for likelihood ratio test. Insignificant predictors (likelihood ratio test p > 0.05) are grayed out. The combined-PI risk predictor had the second largest HR 3.36 (95% CI 1.5—7.4), following TP53 mutation status (HR 3.46 with 95% CI 1.7—7.2). (TIF) [file pone.0017845.s004.tif]

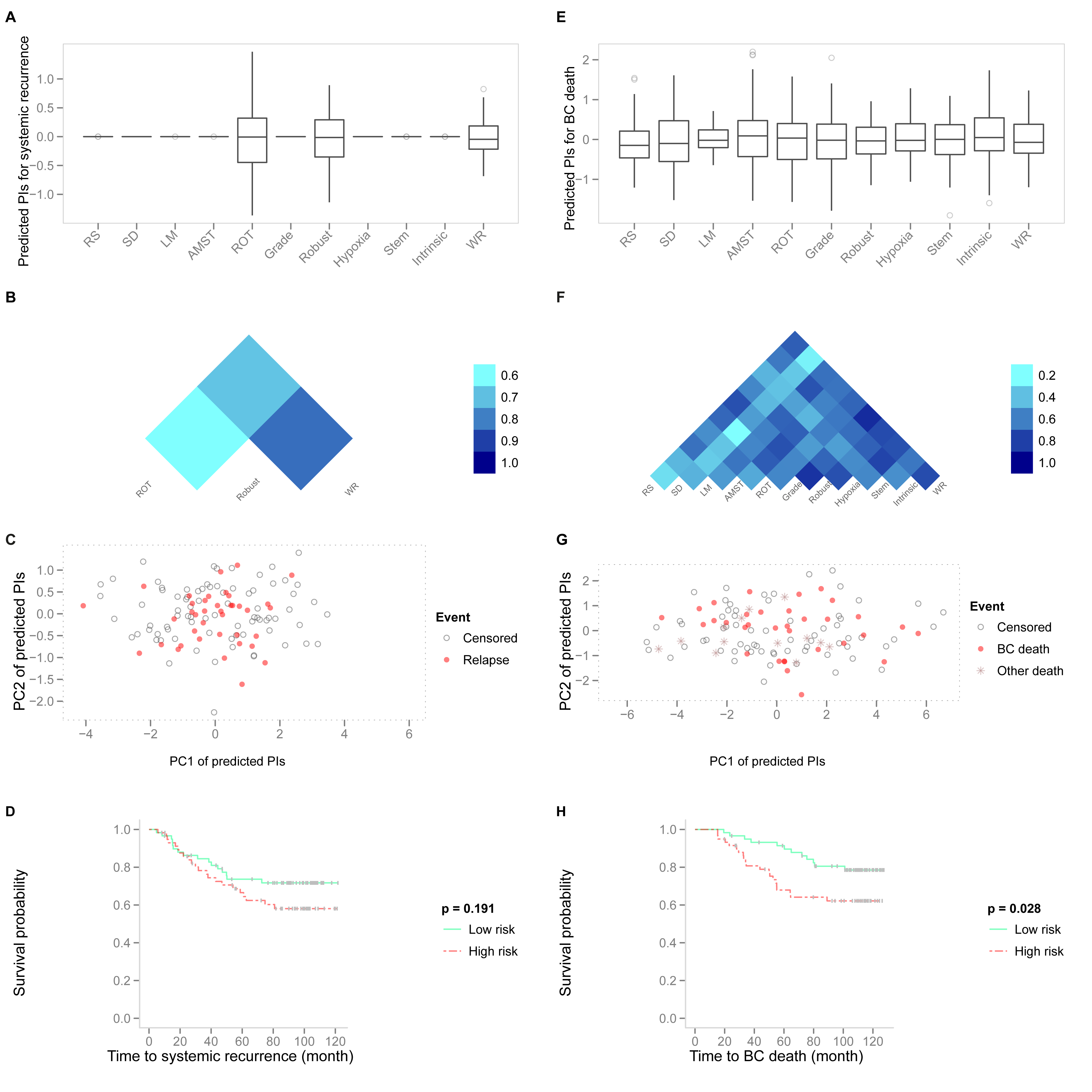

Supplement: Figure S5 — Summary of switched analysis by using Ull as training set and MicMa as test set. Results for systemic recurrence are in (A-D); for BC specific death in (E-H). (A) Boxplot of predicted PI for systemic recurrence. Only three out of eleven gene sets had converged model in model training stage. (B) Spearman correlation structure among gene sets with convergence. (C) Projection of the predicted PIs on space formed by PC1 (captured 81% variability) and PC2. (D) Kaplan-Meier curves associated with the two risk groups by median-cut of PC1 value (logrank p = 0.191). (E) Boxplot of predicted PI for BC specific death. (F) Spearman correlation structure among gene sets with convergence. (G) Projection of the predicted PIs on space formed by PC1 (captured 65% variability) and PC2. (H) Kaplan-Meier curves associated with the two risk groups by median-cut of PC1 value (logrank p = 0.028). (TIF) [file pone.0017845.s005.tif]

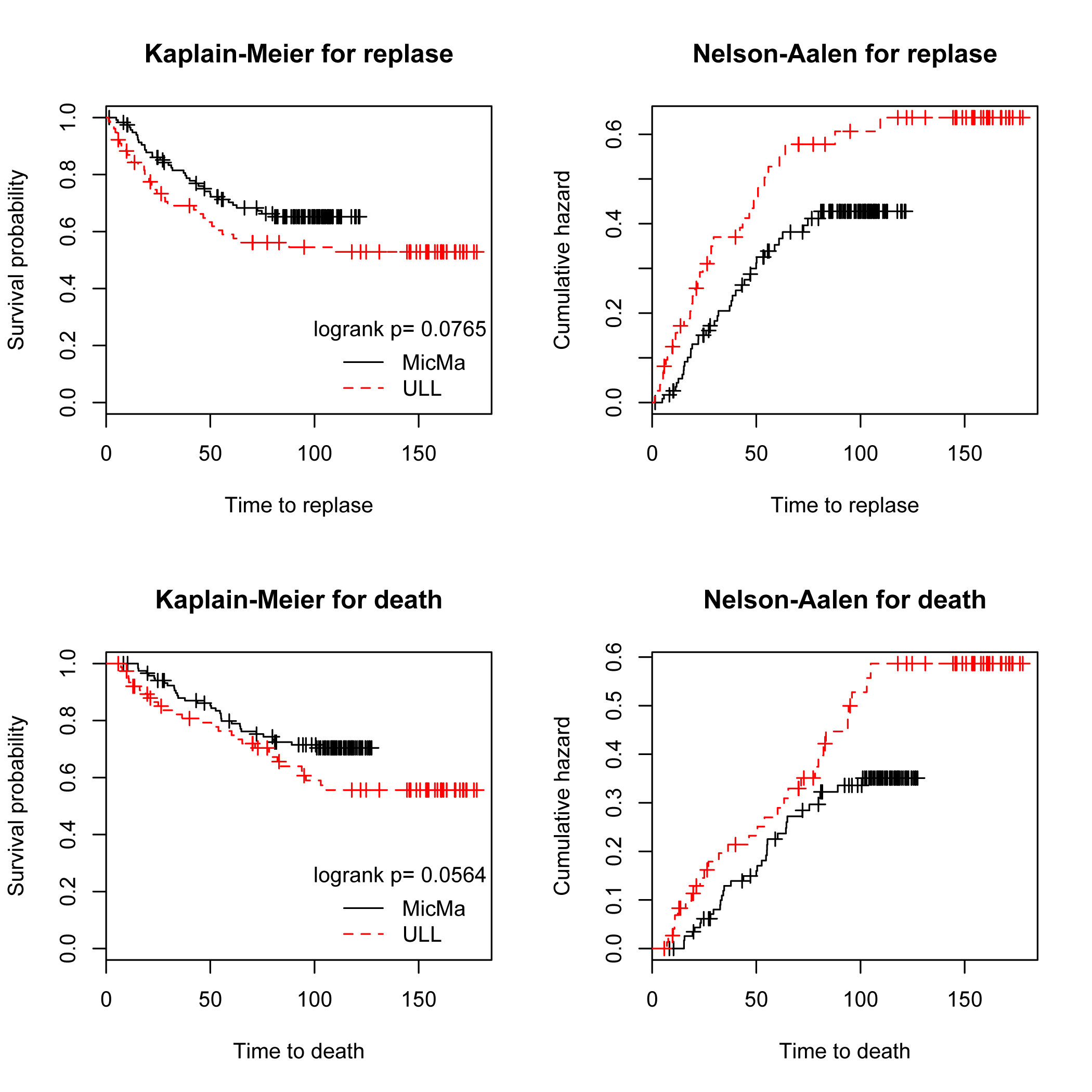

Supplement: Figure S6 — Survival curves for training set (MicMa) and test set (Ull). The logrank test showed that the training cohort and test cohort had borderline significant survival curves for the survival endpoint. (TIF) [file pone.0017845.s006.tif]
