# Supplementary material for: Combining Gene Signatures Improves Prediction of Breast Cancer Survival
Source: PLoS One. 2011 Mar 10;6(3):e17845. doi: 10.1371/journal.pone.0017845 (PMC3053398; doi:10.1371/journal.pone.0017845)
Supplement: Table S1 — Results summary of PCA on predicted PIs for systemic recurrence from 9 converged gene sets. (PDF) [file pone.0017845.s007.pdf]

Table S1. Results summary of PCA on predicted PIs for systemic recurrence from 9 converged gene sets.

Standard deviations:
2.41 1.04 0.85 0.68 0.61 0.49 0.43 0.33 0.15

Rotation:

	PC1	PC2	PC3	PC4	PC5	PC6	PC7	PC8	PC9
RS	0.32	-0.46	0.18	0.02	0.18	-0.55	0.54	-0.15	-0.08
AMST	0.34	-0.11	0.04	-0.46	-0.72	0.28	0.24	0.04	0.02
ROT	0.26	0.46	-0.53	-0.49	0.36	-0.14	0.20	0.13	-0.03
Grade	0.38	-0.28	0.05	-0.02	0.36	0.37	-0.11	0.07	0.70
Robust	0.38	-0.26	0.06	-0.02	0.28	0.37	-0.24	0.15	-0.70
Hypoxia	0.19	0.55	0.79	-0.11	0.13	-0.03	0.05	0.08	0.01
Stem	0.32	0.33	-0.20	0.67	-0.09	0.31	0.41	-0.18	-0.03
Intrinsic	0.38	0.05	-0.09	0.31	-0.27	-0.43	-0.32	0.63	0.08
WR	0.39	0.11	-0.06	-0.04	-0.12	-0.24	-0.51	-0.71	0.01

Importance of components:

	PC1	PC2	PC3	PC4	PC5	PC6	PC7	PC8	PC9
Standard deviation	2.41	1.04	0.85	0.68	0.61	0.49	0.43	0.33	0.15
Proportion of Variance	0.64	0.12	0.08	0.05	0.04	0.03	0.02	0.01	0.00
Cumulative Proportion	0.64	0.76	0.85	0.90	0.94	0.97	0.99	1.00	1.00

Projected scores of test patients on PCs:

	PC1	PC2	PC3	PC4	PC5	PC6	PC7	PC8	PC9
ULL_002	-0.51	0.51	-0.59	-0.11	-0.07	0.58	0.25	0.36	0.22
ULL_007	-0.84	-0.20	-0.71	-0.19	0.17	0.46	-0.13	-0.05	-0.05
ULL_011	2.46	1.23	-0.78	-0.56	0.20	-0.12	-0.21	-0.28	-0.02
ULL_013	-0.68	0.83	-0.41	0.10	-1.02	-0.57	0.88	0.02	-0.02
ULL_014	-0.94	0.49	0.89	0.15	0.29	0.18	-0.36	-0.23	0.03
ULL_016	-0.03	0.20	-0.09	1.42	0.98	0.06	0.43	-0.23	-0.01
ULL_019	0.76	-0.19	-1.53	1.11	0.44	-0.10	0.60	-0.84	0.32
ULL_020	-0.17	0.71	-0.77	-0.64	0.37	-0.39	-0.28	0.01	-0.04
ULL_022	-0.94	-0.62	-0.93	0.98	-0.12	0.33	-0.19	0.20	-0.13
ULL_023	2.72	-0.44	0.11	-0.91	0.12	-0.31	0.62	-0.59	-0.06
ULL_024	-0.76	0.36	-0.93	-1.07	-1.08	0.39	0.44	0.06	-0.02
ULL_026	3.68	0.61	-0.52	-0.21	0.14	-0.11	0.31	0.17	0.17
ULL_027	0.28	0.72	0.28	0.66	-0.76	0.50	-0.22	0.17	-0.08
ULL_028	-0.15	-0.61	-0.83	0.11	-0.95	0.36	0.00	0.16	-0.02
ULL_031	-1.08	2.19	-0.14	1.40	-0.68	0.43	0.69	-0.27	0.07
ULL_036	-2.85	-0.70	0.87	-1.57	0.17	0.11	0.34	-0.19	-0.16
ULL_037	-3.27	-1.28	-0.65	-0.13	0.31	-0.21	-0.15	0.30	0.21
ULL_038	2.23	-0.02	0.21	1.12	-0.03	0.32	-0.45	0.06	0.10
ULL_044	-1.65	-2.66	-0.43	0.40	-0.30	1.09	0.01	0.31	0.02
ULL_046	-1.30	0.24	1.11	-0.54	0.45	0.14	0.65	0.78	-0.30
ULL_048	-1.35	0.20	0.45	0.59	-0.49	0.06	-0.03	0.42	-0.28
ULL_053	1.15	-1.63	1.14	0.86	0.01	-0.17	0.59	0.38	-0.16
ULL_055	-3.52	0.86	0.02	0.08	-0.33	-0.81	-0.47	0.19	0.05
ULL_056	2.21	1.91	1.91	-1.32	0.48	0.44	-0.38	-0.25	-0.02
ULL_057	3.55	-0.20	-0.71	0.26	0.67	0.19	-0.69	-0.04	-0.10
ULL_060	-0.53	1.60	-0.66	0.73	0.64	-0.02	-0.89	-0.34	-0.15
ULL_062	-3.91	1.31	0.78	-0.84	-0.41	-0.23	0.38	-0.59	-0.06
ULL_063	-2.47	-0.47	-2.59	0.29	0.38	-0.08	-0.21	0.31	-0.04
ULL_065	5.74	1.50	0.86	-0.26	-0.78	0.66	-0.26	0.04	0.15
ULL_066	1.21	0.03	0.56	-0.34	1.02	0.44	-0.50	0.16	-0.17
ULL_067	0.24	-2.36	0.56	0.74	-0.34	0.60	0.12	-0.22	-0.07
ULL_069	0.10	-0.27	1.22	-0.88	0.68	0.56	-0.27	0.13	0.00

ULL_071	4.00	-1.21	-0.92	0.39	0.41	-1.10	0.45	-0.03	-0.15
ULL_072	1.16	0.02	-0.27	0.24	0.21	0.18	-0.28	-0.39	-0.01
ULL_074	-3.21	0.98	-0.10	0.58	-0.45	0.03	-0.19	-0.25	-0.23
ULL_075	0.89	-1.42	1.15	0.60	0.28	-0.47	-0.30	0.21	0.15
ULL_079	-2.59	0.72	-0.56	0.07	-0.26	1.46	-0.29	-0.07	-0.22
ULL_080	3.34	0.93	0.42	0.47	1.27	-0.31	-0.03	0.10	0.22
ULL_083	4.23	-1.92	-0.38	-0.03	-1.17	-0.99	-0.06	-0.48	-0.14
ULL_085	-0.57	-0.49	0.18	-0.02	-0.77	-0.13	0.36	-0.07	-0.09
ULL_087	2.04	0.84	1.63	0.24	0.61	-0.52	0.44	-0.56	-0.25
ULL_088	-0.69	1.58	0.75	0.69	-0.26	0.44	0.72	0.62	0.00
ULL_096	4.97	-0.71	0.32	-0.33	-0.92	-0.01	0.13	0.10	-0.33
ULL_097	-2.57	0.47	-0.28	0.31	-0.30	0.32	0.45	-0.21	-0.18
ULL_099	0.78	-1.07	0.02	0.33	0.30	0.54	0.13	0.81	0.23
ULL_101	2.79	0.45	0.30	-0.24	-0.82	-0.34	-0.58	0.22	0.36
ULL_105	0.09	-0.55	0.33	-0.67	-0.93	-0.29	0.02	0.25	-0.09
ULL_107	-0.98	-0.17	-0.67	-0.58	-0.84	-0.08	-0.55	-0.26	0.09
ULL_111	-1.78	-0.06	0.52	-1.12	0.90	0.36	-0.12	-0.44	-0.07
ULL_112	-3.14	1.32	0.06	-1.15	-0.23	0.20	-0.55	-0.01	0.19
ULL_113	2.34	0.19	-0.60	0.83	0.50	0.33	-0.77	-0.29	0.05
ULL_122	-0.48	0.92	-1.01	-0.44	-0.23	0.15	-0.33	0.16	0.01
ULL_123	0.71	-0.21	-1.35	-0.27	-0.62	0.79	0.86	-0.13	0.18
ULL_132	2.06	1.22	-0.15	0.52	-0.57	-0.64	-0.99	0.11	-0.07
ULL_134	-0.80	0.72	-0.62	-0.26	-0.57	-0.85	0.02	0.46	-0.04
ULL_135	-1.53	-0.74	0.55	-0.22	-0.33	-0.60	0.16	-0.31	0.09
ULL_136	-1.19	1.43	0.06	1.32	-0.24	0.16	0.52	-0.19	0.10
ULL_138	-2.30	-1.91	1.85	0.16	-0.12	-0.06	-0.27	-0.23	0.18
ULL_139	0.66	0.02	1.07	-0.79	0.41	0.17	0.74	-0.13	0.43
ULL_143	1.32	0.27	-0.01	-0.54	-0.22	0.12	-0.36	0.38	0.19
ULL_144	-1.85	0.36	-0.26	-0.08	-0.63	-0.47	-0.19	-0.29	0.10
ULL_150	-2.26	-0.77	-0.55	-0.90	0.36	0.21	-0.05	-0.42	0.03
ULL_165	2.26	-0.03	-0.33	0.35	-0.43	0.90	0.07	-0.57	-0.15
ULL_167	2.99	0.56	-1.05	-0.68	1.86	-0.24	0.46	0.21	-0.20
ULL_168	-2.85	0.19	-1.32	-0.71	0.38	-0.25	0.15	-0.08	-0.10
ULL_169	3.03	-0.13	0.19	1.05	-0.02	-1.08	0.36	-0.02	-0.01
ULL_176	-3.64	0.18	-0.92	-0.55	0.76	-1.04	-0.17	0.41	-0.15
ULL_177	4.94	-0.14	0.85	0.34	0.08	0.56	-0.48	0.30	-0.20
ULL_181	-0.03	1.99	0.34	-0.48	-0.62	-0.71	0.22	0.44	0.12
ULL_183	-0.45	-2.15	0.17	0.05	0.03	-0.43	-0.74	0.02	0.09
ULL_184	1.63	0.35	0.94	-0.05	-0.33	-0.44	-0.12	-0.19	-0.01
ULL_188	3.39	-1.33	-2.04	-1.05	0.87	0.25	0.35	-0.20	0.09
ULL_190	-2.38	-1.89	0.66	0.14	0.11	0.23	-0.14	-0.28	0.03
ULL_199	-3.17	-1.18	0.66	-0.01	0.44	-0.24	0.04	0.05	0.08
ULL_201	-1.12	0.51	0.52	0.29	1.30	0.30	0.64	0.18	0.11
ULL_202	1.10	0.24	0.95	-0.17	0.42	-0.09	0.39	0.32	0.12
ULL_214	-0.94	-0.54	-0.16	0.17	0.35	-0.19	0.24	0.65	-0.09
ULL_216	-0.68	0.45	-1.01	-0.13	0.17	-0.26	-0.33	0.36	0.06
ULL_222	-3.31	-1.38	0.72	-0.33	-0.09	-0.14	-0.19	-0.19	-0.07
ULL_230	1.14	-1.34	0.60	-0.50	-0.89	-0.04	-0.12	0.01	0.11
DNR_N_AO100	-6.72	0.57	1.06	1.76	0.71	-0.49	-0.33	-0.23	0.08
