# Supplementary material for: Combining Gene Signatures Improves Prediction of Breast Cancer Survival
Source: PLoS One. 2011 Mar 10;6(3):e17845. doi: 10.1371/journal.pone.0017845 (PMC3053398; doi:10.1371/journal.pone.0017845)
Supplement: Table S2 — Results summary of univariate and multivariate analysis of combined PI-risk predictor for systemic recurrence. (PDF) [file pone.0017845.s008.pdf]

Table S2. Results summary of univariate and multivariate analysis of combined PI-risk predictor for systemic recurrence

1. Univariate analysis

Univariate Cox model incorporating the dichotomized combined-PI as predictor

```
coxph(formula = Surv(time, event == 1) ~ combined_PI, data = tmp)

  n= 77, number of events= 34
   (4 observations deleted due to missingness)

                        coef exp(coef) se(coef)     z Pr(>|z|)
combined_PI:high risk 1.0365    2.8194   0.3678 2.819  0.00482 **
---
Signif. codes:  0 '***' 0.001 '**' 0.01 '*' 0.05 '.' 0.1 ' ' 1

                      exp(coef) exp(-coef) lower .95 upper .95
combined_PI:high risk     2.819     0.3547     1.371     5.797

Rsquare= 0.106   (max possible= 0.971 )
Likelihood ratio test= 8.61  on 1 df,   p=0.003341
Wald test            = 7.94  on 1 df,   p=0.004824
Score (logrank) test = 8.67  on 1 df,   p=0.003242
```

Test proportional hazards assumption for individual univariate Cox models reported in the univariate comparison part

```
$combined_PI
                                 rho  chisq    p
       combined_PI:high risk -0.0483 0.0781 0.78
$tumorSize
                             rho   chisq     p
       tumorSizepT2     -0.15344 0.77843 0.378
       tumorSizepT3-pT4  0.00864 0.00253 0.960
       GLOBAL                 NA 2.10350 0.349
$histoGrade
                     rho chisq     p
       histoGrade2 0.210  1.50 0.220
       histoGrade3 0.204  1.42 0.233
       GLOBAL         NA  1.53 0.465
$TP53
                       rho chisq     p
       TP53mutation -0.144 0.661 0.416
$node
                       rho  chisq     p
       nodepN1     -0.0746 0.1871 0.665
       nodepN2-pN3 -0.0398 0.0523 0.819
       nodeOther   -0.2007 1.2908 0.256
       GLOBAL           NA 1.3267 0.723
$ER
                    rho chisq       p
       ERpositive 0.475  6.85 0.00887
$stage
                  rho   chisq     p
       stage2 -0.11936 0.39224 0.531
       stage3 -0.13508 0.49363 0.482
```

```
       stage4 -0.00722 0.00143 0.970
       GLOBAL       NA 1.11034 0.775
$AOL_risk
                  rho  chisq     p
       AOL_risk -0.0275 0.0201 0.887
$RS
              rho    chisq     p
       RS1 -0.00289 0.000281 0.987
$AMST
               rho  chisq     p
       AMST1 -0.0502 0.0836 0.772
$ROT
             rho chisq     p
       ROT1 -0.239  1.87 0.172
$Grade
                rho  chisq     p
       Grade1 -0.0475 0.0746 0.785
$Robust
                rho chisq     p
       Robust1 -0.065 0.139 0.709
$Hypoxia
                 rho chisq      p
       Hypoxia1 -0.354  4.18 0.0409
$Stem
               rho  chisq     p
       Stem1 -0.0384 0.0491 0.825
$Intrinsic
                  rho chisq     p
       Intrinsic1 0.174  1.02 0.312
$WR
            rho chisq     p
       WR1 0.0921 0.286 0.593
```

Summary of univariate comparison

	Deviance	PVE	p	Cindex	HR	HR_lowerCI	HR_upperCI	type
combined_PI	8.611	0.106	0.003	0.746	2.819	1.371	5.797	GeneSet predictor
AOL_risk	2.283	0.033	0.131	0.734	2.629	0.622	11.106	Clinical parameter
TP53	7.470	0.092	0.006	0.759	2.872	1.415	5.830	Clinical parameter
tumorSize	9.358	0.117	0.009	0.706	NA	NA	NA	Clinical parameter
stage	7.501	0.111	0.058	0.698	NA	NA	NA	Clinical parameter
node	6.090	0.076	0.107	0.605	NA	NA	NA	Clinical parameter
ER	0.227	0.003	0.634	0.403	0.838	0.406	1.727	Clinical parameter
histoGrade	1.979	0.025	0.372	0.480	NA	NA	NA	Clinical parameter
RS	4.827	0.061	0.028	0.686	2.146	1.072	4.296	GeneSet predictor
AMST	2.092	0.027	0.148	0.630	1.646	0.838	3.232	GeneSet predictor
ROT	3.591	0.046	0.058	0.686	1.933	0.966	3.865	GeneSet predictor
Grade	6.174	0.077	0.013	0.712	2.377	1.185	4.767	GeneSet predictor
Robust	5.391	0.068	0.020	0.703	2.257	1.114	4.573	GeneSet predictor
Hypoxia	1.060	0.014	0.303	0.626	1.426	0.727	2.795	GeneSet predictor
Stem	0.115	0.001	0.734	0.535	1.124	0.573	2.205	GeneSet predictor
Intrinsic	0.437	0.006	0.508	0.538	1.261	0.631	2.519	GeneSet predictor
WR	3.093	0.039	0.079	0.640	1.835	0.926	3.636	GeneSet predictor

2. Multivariate analysis

Correlation between TP53 & combined-PI predictor

```
      low risk high risk
  WT        38        22
  Mut        2        18

       Fisher's Exact Test for Count Data
p-value = 5.308e-05
alternative hypothesis: true odds ratio is not equal to 1
95 percent confidence interval:
   3.137077 145.691741
sample estimates:
odds ratio
    15.026
```

Full model: Risk = tumor size + Tp53 + combined_PI + strata(ER)

```
                       coef exp(coef) se(coef)    z     p
tumorSize:pT2         1.005      2.73    0.575 1.75 0.080
tumorSize:pT3-pT4     1.200      3.32    0.658 1.82 0.068
TP53:mutation         0.732      2.08    0.575 1.27 0.200
combined_PI:high risk 0.974      2.65    0.468 2.08 0.037

Likelihood ratio test=16.1  on 4 df, p=0.00296  n= 68, number of events= 31 (13 observations
deleted due to missingness)
```

Model 2: Risk = tumor size + Tp53 + strata(ER)

```
                  coef exp(coef) se(coef)    z     p
tumorSize:pT2     1.14      3.14    0.576 1.98 0.047
tumorSize:pT3-pT4 1.43      4.17    0.660 2.16 0.030
TP53:mutation     1.33      3.79    0.519 2.57 0.010

Likelihood ratio test=11.8  on 3 df, p=0.00793  n= 68, number of events= 31 (13 observations
deleted due to missingness)
```

Model comparison by AIC and Analysis of Deviance: Full model vs Model 2

```
> stepAIC(model_full)
Start:  AIC=191.8
Surv(time, event == 1) ~ tumorSize + TP53 + combined_PI + strata(ER)

>  stepAIC(model2)
Start:  AIC=194.01
Surv(time, event == 1) ~ tumorSize + TP53 + strata(ER)

Analysis of Deviance Table
Cox model: response is  Surv(time, event == 1)
 Model 1: ~ tumorSize + TP53 + combined_PI + strata(ER)
 Model 2: ~ tumorSize + TP53 + strata(ER)
   loglik  Chisq Df P(>|Chi|)
1 -91.902
2 -94.003 4.2011  1    0.0404 *
---
Signif. codes:  0 '***' 0.001 '**' 0.01 '*' 0.05 '.' 0.1 ' ' 1
```

Model selection by AIC:

```
stepAIC(model_full, direction = "both")
Start:  AIC=191.8
Surv(time, event == 1) ~ tumorSize + TP53 + combined_PI + strata(ER)
              Df    AIC
- TP53         1 191.46
<none>           191.80
- tumorSize    2 192.25
- combined_PI  1 194.01

Step:  AIC=191.46
Surv(time, event == 1) ~ tumorSize + combined_PI + strata(ER)
              Df    AIC
<none>           191.46
- tumorSize    2 191.51
+ TP53         1 191.80
- combined_PI  1 198.66
Call:
coxph(formula = Surv(time, event == 1) ~ tumorSize + combined_PI + strata(ER), data = tmp)

                      coef exp(coef) se(coef)    z      p
tumorSize:pT2        0.952      2.59    0.563 1.69 0.0910
tumorSize:pT3-pT4    1.090      2.97    0.640 1.70 0.0890
combined_PI:high risk 1.206      3.34    0.413 2.92 0.0035

Likelihood ratio test=14.4  on 3 df, p=0.00241  n= 68, number of events= 31
```

Final model: Risk = tumor size + combined_PI + strata(ER)

```
  n= 68, number of events= 31 (13 observations deleted due to missingness)

                      coef exp(coef) se(coef)     z Pr(>|z|)
tumorSize:pT2        0.9524    2.5920   0.5628 1.692  0.09059 .
tumorSize:pT3-pT4    1.0897    2.9734   0.6398 1.703  0.08853 .
combined_PI:high risk 1.2060    3.3402   0.4132 2.919  0.00351 **
---
Signif. codes:  0 '***' 0.001 '**' 0.01 '*' 0.05 '.' 0.1 ' ' 1

                      exp(coef) exp(-coef) lower .95 upper .95
tumorSize:pT2             2.592     0.3858    0.8602     7.811
tumorSize:pT3-pT4         2.973     0.3363    0.8485    10.420
combined_PI:high risk     3.340     0.2994    1.4862     7.507

Rsquare= 0.191   (max possible= 0.947)
Likelihood ratio test= 14.39  on 3 df,   p=0.002415
Wald test            = 12.9  on 3 df,   p=0.004867
Score (logrank) test = 14.57  on 3 df,   p=0.002222
```

Test proportional hazards assumption for Final model

```
                          rho  chisq     p
tumorSize:pT2         -0.0402 0.0477 0.827
tumorSize:pT3-pT4      0.0378 0.0389 0.844
combined_PI:high risk  0.1143 0.4301 0.512
GLOBAL                     NA 0.7959 0.850
```
