# Supplementary material for: Combining Gene Signatures Improves Prediction of Breast Cancer Survival
Source: PLoS One. 2011 Mar 10;6(3):e17845. doi: 10.1371/journal.pone.0017845 (PMC3053398; doi:10.1371/journal.pone.0017845)
Supplement: Table S3 — Molecular and clinicopathological characteristics of the tumor material in MicMa and Ull datasets. (PDF) [file pone.0017845.s009.pdf]

Table S3. Molecular and clinicopathological characteristics of the tumor material in MicMa and Ull datasets.

		MicMa		Ull	
Characteristics		**n**	**%§**	**n**	**%§**
All		123	100	80*	100
Molecular subtype	Luminal A	50	40.7	23	28.8
	Luminal B	16	13.0	15	18.8
	ERBB2+	21	17.1	10	12.5
	Basal-like	17	13.8	12	15.0
	Normal-like	15	12.2	20	25.0
	Unclassified	4	3.3		
TP53	Mutant	38	32.5	20	25.0
	Wild type	79	67.5	60	75.0
	Missing	6			
Estrogen receptor	Positive	80	66.1	45	62.5
	Negative	41	33.9	27	37.5
	Missing	2		8	
Histological Grade	I	16	13.2	6	7.5
	II	58	47.9	53	66.3
	III	47	38.8	21	26.3
	Missing	2			
Tumor status	pT1 (≤ 2cm)	55	44.7	20	26.0
	pT2 (> 2 ≤ 5cm)	51	41.5	44	57.1
	pT3-4 (> 5cm)	13	10.6	14	18.2
	pTX¤	3	2.4	2	
Nodal status	pN0 (Negative)	50	40.7	33	41.3
	pN1 (1-3 positive)	35	28.5	23	28.8
	pN2-3 (≥ 4 positive)	31	17.1	12	15.0
	pNX#	7	5.7	12	15.0
Systemic relapse	Yes	39	32.5	34	44.2
	No	81	67.5	43	55.8
	Missing	3		3	
Death	From breast cancer	34	27.6	30	39.0
	Other cause	21	17.1	20	26.0
	No	68	55.3	25	32.5
	Emmigrated			2	2.6
	Missing			3	

For all parameters, numbers are calculated excluding missing data§.

§Missing includes both non-evaluable cases, not performed and information not available.

* Ull cohort (n = 81) contains 80 tumor samples and one additional healthy tissue, which was excluded from the calculation.

¤ pTX defines those cases where no determination of tumor size was possible.

pNX defines those cases where no axillary dissection was performed but no clinical sign of nodal metastasis existed.
