# Supplementary material for: Combining Gene Signatures Improves Prediction of Breast Cancer Survival
Source: PLoS One. 2011 Mar 10;6(3):e17845. doi: 10.1371/journal.pone.0017845 (PMC3053398; doi:10.1371/journal.pone.0017845)
Supplement: Methods S1 — Supplementary methods, results, notes, and references. (PDF) [file pone.0017845.s010.pdf]

Supplemental text for

Combining gene signatures improves prediction of breast cancer survival

Xi Zhao, Einar Andreas Rødland, Therese Sørlie, Bjørn Naume, Anita Langerød, Arnoldo Frigessi, Vessela N. Kristensen, Anne-Lise Børresen-Dale, Ole Christian Lingjærde

Contents

I. Materials and Microarray protocol

- Training set (MicMa)
- Validation set (Ull)

II. A brief discussion of the various gene sets & Adjuvant! Online model

III. Cross-platform gene mapping

IV. Statistical analysis

- Theoretical background: Cox regression
- Theoretical background: Cox-ridge regression
- Theoretical background: Model selection by cross-validation
- Construct a gene-set prediction model based on gene expression
- Model evaluation: proportion of variation explained (PVE)
- Model evaluation: concordance index (C-index)

V. Supplementary Results

- Evaluation on the training set for systemic relapse as clinical endpoint
- Results summary: analysis for BC specific death as clinical endpoint
    1. PCA summary for endpoint BC death
    2. Univariate analysis of Combined PI-risk predictor for BC death
    3. Multivariate analysis of Combined PI-risk predictor for BC death

VI. Supplementary References

I: Materials and Microarray protocol

Training set (MicMa)

The published data [1] have 123 human breast cancer cases, mainly stage I and II. Patients treated for localized breast cancer were included in this study [2] between 1995 to 1998. This cohort is a subset of a larger study where the patients were monitored for presence of disseminated tumor cells in bone marrow (DTC). The selection of patients to adjuvant treatment was based upon the prevailing National Guidelines, where postmenopausal hormone receptor (HR) positive patients received tamoxifen only, postmenopausal HR negative patients received CMF (Cyclophosphamide, Methotrexate, Fluorouracil) and premenopausal patients received CMF followed by tamoxifen if HR positive. Five patients received high dose chemotherapy and another five, preoperative chemotherapy due to large tumor size [2]. After completed primary therapy, the patients were followed at 6-12 months intervals. Fresh frozen tissue samples were available from all the 123 individuals. One patient was diagnosed with ductal carcinoma in situ.

Total RNA was isolated from primary tumor tissues using the TRIzol reagent (Invitrogen). The integrity and quality of RNA samples were evaluated on the 2100 Bioanalyzer (Agilent) and the concentration measured using a NanoDrop spectrophotometer (NanoDrop Technologies). Amplification, and labeling of RNA from tumor with Cy5 and from the Universal Human Reference (Stratagene) with Cy3, was performed as previously described [3]. Hybridization of labeled cRNA to arrays containing 42,000 features representing 23169 unique cluster IDs (UniGene Build Number 215) produced at the Stanford Functional Genomics Facility (http://www.microarray.org/sfgf/jsp/home.jsp) was performed at 65°C overnight as previously described. The hybridized arrays were scanned on an Agilent DNA microarray scanner and images analyzed by GenePix Pro v 4.1. All procedures are available at http://genome-www.stanford.edu/breast_cancer and all raw data can be obtained from the Stanford Microarray Database (SMD; http://smd.stanford.edu/). The data have been deposited in NCBIs Gene Expression Omnibus (GEO; http://www.ncbi.nlm.nih.gov/geo/) and are accessible through GEO Series accession number GSE3985.

Validation set (Ull)

The data published by Langerød et. al [4] was used as validation dataset. Patient samples were sequentially collected at Ullevål University Hospital from 1990 to 1994. 80 cases were selected from the total series based only on sufficient amount of fresh frozen tissue for microarray. The last update of patient information was done in 2006, providing an observation time of 12 to 16 years. Patients were followed until death or emigration, and only 12 patients were lost to follow-up. The average age of the 80 cases analyzed by cDNA microarrays was 65.0 years at time of primary surgery (range 28.2 to 87.7 years).

Total RNA was isolated from snap frozen tumor tissue using TRIzol® solution (Invitrogen™, Carlsbad, California, USA). The concentration of total RNA was determined using an HP 8453 spectrophotometer (Hewlett Packard) and the integrity of the RNA was assessed using a 2100 Bioanalyzer (Agilent, Santa Clara, California, USA). Linear amplification of total RNA was performed using an optimized protocol previously described [3]. Amplified tumor RNA was

labeled by Cy5 and amplified RNA from Universal Human Reference total RNA (Stratagene®, La Jolla, California, USA) was labeled by Cy3. The labeling and hybridization of amplified RNA to cDNA microarrays, containing more than 42,000 elements, was performed as previously described [3]. Experimental protocols can be found at the referred web site (http://www.stanford.edu/group/sjeffreylab/). Images of the arrays was acquired using a Gene Pix 4000B scanner (Axon Instruments, Sunnyvale, California, USA), and analyzed using Gene Pix Pro 3.0/4.0/4.1 software and by visual inspection. Data were entered in the Stanford Microarray Database (http://smd.stanford.edu/).

II: A brief discussion of the various gene sets & Adjuvant! Online model

Except for the RS predictor, all gene sets were originally developed as microarray-based predictors.

1. 16-gene recurrence-score predictor (RS)

   These 16 genes are the cancer-related subset from the 21-gene recurrence score RT-PCR-based assay (Oncotype DX®) in Paik *et al.* (2004) [5]. The expression for each cancer-related gene on RT-PCR assay is normalized relative to the expression of the five reference genes, and further used to calculate a recurrence score, *RS*, which quantifies the likelihood of distant recurrence in adjuvant-tamoxifen-treated patients with lymph node-negative, ER-positive breast cancer into categories of high risk ($RS \geq 31$), intermediate risk ($18 \leq RS < 31$), and low risk of recurrence ($RS < 18$). They claimed Recurrence Score® performance is superior to that of patient age, tumor size or tumor grade in either predictive power or reproducibility.

2. 26-gene stroma-derived prognostic predictor (SD)

   This gene set was identified from an analysis of microdissected stroma from breast cancer specimens. A logistic regression was used to score and rank each gene in the expression profile on the basis of its statistical significance in predicting recurrence in a model that included the gene expression level, lymph node status, and ER, PR and HER2 status. And naive Bayes' classifiers were trained to predict outcome using the ranked gene expression profile of the recurrence-positive stroma cluster. The original study [6] reported that 26-SD prognostic predictor stratified disease outcome independently of standard clinical prognostic factors and published expression-based predictors.

3. 54-lung-metastasis-gene signature (LM)

   By means of in vivo selection, transcriptomic analysis, functional verification and clinical validation, a set of 54 genes was identified that mediates risk of breast cancer metastasis to lung and is clinically correlated with the development of lung metastasis when expressed in primary breast cancers [7]. A cohort of 82 breast cancer patients was used in a univariate Cox proportional hazards model to relate the expression level of each lung metastasis signature gene with clinical outcome. A 'leave-one-out' cross-validated multivariate analysis was used to distinguish between patients with a high risk and those with a low risk for developing lung metastasis. In each round, each of the 54 gene weights was estimated in a univariate Cox using only training set, and a 10-year lung-metastasis risk index for test case (held out from the full set of tumors) was defined as a linear combination of gene expression values weighted by their univariate result; and the held out tumor was assigned to the high- or low-risk groups. The 20th percentile of the risk index scores was chosen as the threshold to separate high risk (top 20%) and low risk group.

4. 70-gene predictor (AMST)

This breast cancer prognostic signature [8], also known as MammaPrint® genomic test, consists of 70 most informative genes derived from a supervised statistical model. Based on the expression levels of the 70 genes, it predicts distant metastases status 5 years after the diagnosis on younger patients with stage I and II node-negative breast cancer. The signature associated with a poor prognosis demonstrates overexpression of genes regulating the cell cycle, invasion, metastasis, and angiogenesis. In 2007, MammaPrint® test was approved by FDA for use in the U.S. for lymph node negative breast cancer patients under 61 years of age with tumors of less than 5cm. It is the first cleared molecular test that profiles genetic activity.

In a recent preliminary clinical trial, Mook *et al.* (2008) [9] reported that the 70-gene prognosis-signature outperforms traditional prognostic factors in predicting metastasis in patients with 1–3 positive nodes. According to the authors, the signature can accurately identify patients with a good disease outcome in node-positive breast cancer, who would not benefit from adjuvant chemotherapy.

5. 76-gene predictor (ROT)

This gene set was used to predict development of distant metastases within 5 years. It was developed from 286 patients not receiving systemic treatment, with lymph node negative primary breast cancer of variable size and from all age groups [10].

6. 97-grade-associated markers (Grade)

Sotiriou *et al.* (2006) [11] identified 97 grade-associated genes from 64 ER positive tumor samples by comparing expression profiles between histologic grade 3 and grade 1 tumors. The signed sum of gene expression values of these 97 genes were used to calculate the gene expression grade index (GGI). In validation datasets, the GGI was strongly associated with histologic grade.

7. 127-gene classifier (Robust)

127 genes were identified as predictors of prognosis from a pooled dataset consisting of six breast cancer datasets, totaling 947 samples [12]. The study showed a significant positive correlation between the number of datasets that is pooled, the validation performance, the number of genes selected, and the enrichment of specific functional categories. The authors claimed that the 127 gene signature is one of the most robust signatures currently available.

8. 168-hypoxia-gene signature (Hypoxia)

The epithelial hypoxia signature [13] consists of 168 genes that were consistently induced by hypoxia. They were defined from an in vitro experiment by Chi *et al.* (2006) [13] where four epithelial cell lines were exposed to hypoxia. A gene expression classier was developed through a "hypoxia score" for the patient by averaging expression levels for the hypoxia-response genes. Patients were assigned into high or low hypoxia response group by a cutoff

hypoxia-score at zero. Using published data sets, the authors found that the “high hypoxia response” group tends to be higher grade, and more likely to have p53 and oestrogen-receptor deficiencies, and, most importantly, a significant association with a poorer prognosis in breast and ovarian cancer.

9. 186-invasiveness-gene signature (Stem)

   Liu *et al.* (2007) [14] report on a 186-gene “invasiveness” gene signature (IGS) that discriminates between normal breast epithelium and tumorigenic breast-cancer cells that are characterized by CD44 expression and low or undetectable levels of CD24. This signature was reported to be associated with survival among patients with breast cancer.

10. 306-intrinsic/UNC gene list (Intrinsic)

   The original intrinsic gene list was previously identified using DNA microarray in the study of Perou *et al*. (2000) [15] and refined in Sørlie *et al*. (2001) [16]. It has been used to identify five distinct subtypes of breast tumors (basal-like, ERBB2+, luminal A, luminal B and normal-breast like) based on the expression profile of the ~ 500 *intrinsic genes*, whose variation was significantly greater between samples from different tumors than between samples from the same tumor before and after treatment. These subtypes were associated with significant differences in overall and disease-free survival [16,17]. The intrinsic/UNC gene list [18] is a new intrinsic-subtype classifier, which uses gene expression profiles to distinguish among breast cancers on the basis of either their cell type of origin — the luminal cell (which is ER-positive) or the basal cell (which lacks expression of ER, the progesterone receptor, and HER2) — or whether the tumor is HER2-positive. Among the UNC intrinsic genes, a proliferation signature was not present in previous breast intrinsic gene sets.

11. 512-core serum response gene list (WR)

   The wound response/core serum response gene list [19] based on a wound-response gene-expression signature derived from the transcriptional response of normal fibroblasts to serum in cell culture, has been shown to improve the risk stratification of early breast cancer over that provided by standard clinic pathological features, in that the development of distant metastases is more likely among patients whose breast cancers have activated pathways for matrix remodeling, cell motility, and angiogenesis than among those whose cancers do not.

12. Adjuvant! Online model (AOL)

   Adjuvant! Online [20] is a computer based model using patient age, comorbidity level, ER status, tumor grade, tumor size and number of positive lymph nodes to predict breast cancer specific mortality and recurrence risk, as well as the benefit of adjuvant therapy for women with early-stage breast cancer.

   Because Adjuvant! was directly derived from mortality data and because details of local therapy (sugery and initial radiation) can strongly influence local relapse rates more so than

mortality, Adjuvant!'s estimates of mortality are more firmly based than those for relapse. Breast cancer outcome estimates made by Adjuvant! are for “patients who have unilateral, unicentric, invasive adenocarcinoma of the breast, who have undergone definitive primary breast surgery and axillary node staging, and who have no evidence of metastatic or known residual disease; no evidence of T4 features (extension to skin or chest wall); no evidence of inflammatory breast cancer. If they have had breast conserving therapy there should be plans for them to receive radiation therapy. They should not yet have received systemic therapy (neoadjuvant therapy), or radiation prior to their surgical staging.” (Adjuvant! Breast Cancer Help Files).

To calculate the 10-year mortality risk for the 80 patients in the test data (Ull), 3 patients with NEOadjuvant; 2 additional patients with metastasis; 3 additional patients with T4 were excluded. Among the rest 72 patients, 6 patients are with “other” histologic subtypes. Due to the differences between the coding used in Adjuvant! Online and in Ull set for node status and tumor size, "positive node = 0" in AOL was specified for Ull patients with unknown node status; “tumor size = 0.1-1.0cm” in AOL was specified for patients with unknown tumor size; “tumor size = 1.1-2.0cm” was specified for patients with T1 (<=2cm), “tumor size = 3.1-5.0cm” was specified for Ull patients with T2 (>2 & <=5cm). The default level “Minor Problems” was kept for comorbidity in AOL to give an estimate of the general health of an individual.

III. Cross-platform gene mapping

The annotations for the Stanford 43k clones were retrieved from SMD (http://smd.stanford.edu/; UniGene Build Number 215). For gene sets that developed from the same platform as Stanford 43k cDNA array, we matched the genes in the gene set to MicMa and Ull through clone IDs. For those developed from other platforms, the matching was done by first using UniGene ID and gene symbols to link to Stanford 43k Clone IDs; and then using gene aliases for those genes found no match in the preceding step. See table 1 for an example.

Table 1: Illustration of matching gene set to Stanford 43k array. ***Step 1***, UniGene IDs for all the probes were retrieved (column "Linker ID 1"). Probe 1 and 2 were found to share the same UniGene IDs with Stanford 43k array clone 566115 and 310356/69002, respectively. ***Step 2***, gene symbols were retrieved for probes 3, 4, 5 and 6 (column "Linker ID 2") that had no match in step 1. Probe 3 and 4 were matched to clone 122274 and 358030, respectively, linking through shared gene symbols. ***Step 3***, gene aliases were retrieved for probe 5 and 6 (column "Linker ID 3") that had no match after step 1 and 2. Probe 5 was matched to clone 33045 by common gene aliases. There is no corresponding clone on Stanford 43k array for probe 6.

Procedure	Probe	Original ID	Linker ID 1<br>*UniGene*	Linker ID 2<br>*Gene symbol*	Linker ID 3<br>*Gene aliases*	Matched Clone ID
Step 1	1	214827_at	Hs.589848			566115
	2	221009_s_at	Hs.9613			310356<br>69002
Step 2	3	202728_s_at	Hs.619315	LTBP1		122274
	4	203571_s_at	Hs.700589	C10orf116		358030
Step 3	5	217028_at	Hs.593413	CXCR4	NPYR	33045
	6	219959_at	Hs.405028	MOCOS	MOS	No match

* Unigene IDs and gene symbols for the genes in the gene sets were retrieved either from manufacture chip annotation files or from SMD database (UniGene Build Number 215).

Furthermore for the matched clones representing the same gene symbol were mean-averaged. For clones without information of gene symbol, we mean-averaged the multiple matched clones that represent the original probe IDs from the corresponding gene signature.

Details of the matching results for individual gene set are describes as follows.

1. RS (16-gene-recurrence-score predictor):

   Original ID: 16 gene symbols were published.

   Retrieved ID: Corresponding *UniGene IDs* were retrieved using gene symbols from SMD (Build #215).

   Results: 13 matches were found on Stanford 43k array using gene symbols. For those didn't find match by symbol, additional 2 matches were found using gene aliases.

   Total: 15/16 = 94%

2. SD (26-gene stroma-derived prognostic predictor):

   Original ID: 26 gene symbols were published.

   Retrieved ID: Corresponding UniGene IDs were retrieved using gene symbols from SMD (Build #215).

   Results: 21 matches were found on Stanford 43k array by symbols. For those didn't find match by symbol, additional 1 match was found by using retrieved UniGeneID.

   Total: 22/26 = 85%

3. LM (54-lung-metastasis-gene signature):

   Original ID: 54 Affy probes were published.

   Retrieved ID: Corresponding UG & symbols were retrieved using 54 Affy probes from chip annotation file for Affy U133A chip.

   Results: 45 matches were found on Stanford 43k array using UniGeneID. For those didn't find match by UniGene, additional 2 matches were found using symbols. For those still didn't find match, additional 1 match was found by using gene aliases.

   Total: 48/54 = 89%

4. AMST (70-gene predictor):

   Original ID: 70 sequence IDs (including EST & Genbank ID) were published.

   Retrieved ID: 70 Genbank IDs were retrieved from the sequence IDs using the published file (http://www.rii.com/publications/2002/vantveer.html). Corresponding UniGeneIDs & gene symbols were retrieved by Genbank IDs from SMD (build #215).

   Results: 53 matches were found on Stanford 43k array using UniGeneID. For those didn't find match, additional 4 matches were found using gene aliases.

   Total: 57/70=81%

5. ROT (76-gene predictor):

   Original ID: 76 Affy probes were published.

   Retrieved ID: Corresponding UniGeneIDs & gene symbols were retrieved using Affy probes from chip annotation file for Affy U133A chip.

   Results: 59 matches were found on Stanford 43k array using UniGeneID. For those didn't find match by UniGene, additional 3 matches were found using symbols. For those still didn't find match, additional 4 matches were found by using gene aliases.

   Total: 66/76=87%

6. Grade (97- histologic-grade-associated markers):

   Original ID: 128 Affy probes were published.

   Retrieved ID: Corresponding UniGeneIDs & gene symbols were retrieved using Affy probes from chip annotation file for Affy U133A chip.

   Results: 101 matches were found on Stanford 43k array using UniGeneID. For those didn't find match by UniGene, additional 6 matches were found using symbols. For those still didn't find match, additional 4 matches were found by using gene aliases.

   Total: 111/128=87%

7. Robust (127-gene classifier):

   Original ID: 127 Affy probes were published.

   Retrieved ID: Corresponding UniGeneIDs & gene symbols were retrieved using Affy probes from chip annotation file for Affy U133A chip.

   Results: 108 matches were found on Stanford 43k array using UniGeneID. For those didn't find match by UniGene, additional 6 matches were found using symbols.

   Total: 114/127=90%

8. Hypoxia (168-hypoxia-gene signature):

   Original ID: 253 CloneIDs were published.

   Results: all 253 matches were found on Stanford 43k array using CLID.

   Total: 253/253 = 100%

9. Stem (186-invasiveness-gene signature):

   Original ID: 186 Affy probeIDs were published.

   Retrieved ID: Since the probes were from different arrays, and information about which probes came from which array were not published, 186 gene symbols were retrieved from the published annotation file (http://content.nejm.org/content/vol356/issue3/images/data/217/DC1/NEJM_Liu_217sa2.xls); and then UniGeneIDs were retrieved by gene symbols from (build#215), further used for the mapping.

   Results: 109 matches were found on Stanford 43k array using gene symbol. For those didn't find match, additional 52 matches were found using gene aliases.

   Total: 161/186=87%

10. Intrinsic (306-intrinsic/UNC gene list):

Original ID: 327 Agilent probe IDs were published.

Retrieved ID: Since the 327 probes were from different arrays, and information about which probes came from which array were not published. Corresponding UniGeneIDs & gene symbols for 327 probes were retrieved from the published 1500-probes-Annotation-table (https://genome.unc.edu/pubsup/breastTumor/Hu-et-al-Intrinsic-List-with-Annotation.xls).

*304 EntrezIDs were also provided by UNC author. There are 294 overlapping with published-EntrezIDs. 290 out of 304 UNC-EntrezIDs can be mapped on Stanford43k. Did not achieve 100% match (according to UNC, it should be 100% match using Entrez alone). Therefore, we used UG & symbols & aliases for the mapping instead of Entrez.

Results: 111 probes were mapped on Stanford 43k array by using UniGene; additional 157 probes were found by using gene symbol; additional 22 probes by gene aliases.

Total: 290/306=95%

11. WR (512 wound response/core serum response gene list):

    Original ID: 573 Clone IDs were published

    Results: 573 matches were found on Stanford 43k array using CLID. (Although the platforms are the same, it's possible some clones were removed from the newer version of the Stanford 43k array.

    Total: 561/573=98%

IV. Statistical analysis

A total 118 patients with available information of systemic recurrence status and time to recurrence (n = 118) were considered in the analysis. For breast cancer specific survival, information was available for 123 patients (n = 123), and these were used in the further analyses.

Theoretical background: Cox regression

The Cox proportional hazards model [21] is widely used to model survival data. No assumption has to be made for the underlying parametric distribution of survival times. We applied this technique to model and predict our time-to-event data. We aimed to predict survival probability for systemic recurrence and breast cancer specific death, respectively. The variables included in the model were expressions of individual genes from gene sets and also the different clinical parameters in the later stage of the analysis.

Our data are (t_j, d_j, $\mathbf{X}_j$), j = 1,…, N, where t_j is the time on study of the jth patient, d_j is an indicator with $d_j = 1$ indicating an event (systemic recurrence or breast cancer specific death) and $d_j = 0$ indicating no event at the observed time, and $\mathbf{X}_j$ is the vector of risk variables associated with the jth patient. In a Cox proportional hazards model, the hazard rate for the jth individual with risk vector $\mathbf{X}_j$ at time t is as follows:

$$h(t \mid \mathbf{X}_j) = h_0(t)\exp(\underline{\beta}^T \mathbf{X}_j) \quad (1)$$

Here, $\underline{\beta}$ is a parameter vector to be estimated and $h_0(t)$ is an arbitrary baseline hazard function. Even though the form of the baseline hazard function is unspecified, coefficients for the risk vector in the Cox model can still be estimated by maximizing a partial likelihood [21]:

$$l(\beta) = \sum_{j=1}^{N} d_j(\underline{\beta}^T \mathbf{X}_j - \ln(\sum_{h \in R_j} \exp(\underline{\beta}^T \mathbf{X}_h))) \quad (2)$$

where R_j is the set of individuals still alive and uncensored just before time t_j.

Theoretical background: Cox-ridge regression

In our case, the number of explanatory variables genes (p) is large and sometimes even exceeds the number of individuals N used for training of the Cox model. Thus overfitting may easily arise, leading to a fitted model that describes that training data well, but performs poorly on prediction of new observations. In addition, a high degree of collinearity among the variables is likely to emerge, thereby leading to a situation in which the estimated regression coefficients may change substantially even after slight perturbations of the training data.

In a linear model, dimension reduction techniques and penalized regression are the strategies to control and stabilize the variance of the estimates and further achieve better prediction rules. Some widely used regression regularization methods such as ridge regression, partial least squares and principle components regression were compared in the study by Frank and Friedman in 1993 [22]. In Cox-ridge regression, the coefficients are estimated by maximization of the penalized partial log-likelihood (using Newton-Raphson procedure):

$$l_\lambda(\beta) = l(\beta) - \frac{1}{2}\lambda \sum_{i=1}^{p} \beta_i^2 \quad (3)$$

where i =1, …, p, indicates the covariates; the first term is the partial log-likelihood and the second term is the penalty term [23]. Here, $\lambda>0$ is a tuning parameter that controls how much weight to put on the penalty function.

Theoretical background: Model selection by cross-validation

The ridge regression estimates depends on the tuning parameter λ. In our study, λ was determined by maximizing the cross-validated partial log-likelihood criterion proposed by Verweij and van Houwelingen (1993) [24]. Let $l^{(-i)}(\beta)$ denote the partial log-likelihood when observation i is left out (i = 1,…, n), $l^{(-i)}(\beta) = \sum_{j \neq i} d_j (\underline{\beta}^T \underline{X}_j - \ln(\sum_{h \in R_j} \exp(\underline{\beta}^T \underline{X}_h)))$. Then, the contribution of observation i to the likelihood is $l^i(\beta) = l(\beta) - l^{(-i)}(\beta)$. Let $\hat{\beta}^{(-i)}$ be the value of β that maximizes $l^{(-i)}(\beta)$. Then the leave-one-out cross-validated partial log-likelihood criterion proposed by Verweij and van Houwelingen (1993) [24] is given by:

$$cvl(\lambda) = \sum_{i=1}^{n} \left\{ l(\hat{\beta}_\lambda^{(-i)}) - l^{(-i)}(\hat{\beta}_\lambda^{(-i)}) \right\} \quad (4)$$

Maximizing the cross-validated partial log-likelihood with respect to λ yields the optimal value for the tuning parameter.

Note that in this paper, we focus on obtaining a prediction rule that performs well on new observations. Therefore, the value λ is determined by maximizing the predictive value of the model. In parametric models [25], the measure for the prediction error is to use the predictive log-likelihood, which is the expected value of the model log-likelihood for new observations. Due to the semi-parametric nature of Cox model, the partial log-likelihood instead of the full log-likelihood is used as a predictive log-likelihood so that the baseline hazard does not play a role.

In a comparative study of survival prediction performance using microarray data [26], it has been found that Cox-ridge regression often outperforms other common regularization techniques for Cox regression, such as principal components regression, supervised principal components regression, partial least squares regression and the lasso.

Construct a gee-set prediction model based on gene expression

On the training set, we apply leave-one-out cross validation using Van Houwelingen's criteria [24] to determine the tuning parameter $\hat{\lambda}$, which is specific for each gene set. We then built a Cox-ridge model using $\hat{\lambda}$ for each gene set by incorporating the intensity log ratio of genes from the gene set as covariates. Let the vector of gene expression values for the ith patient and the jth

gene set be: $\mathbf{X}_{ij} = (X_{i1}, X_{i2}, ..., X_{i,n_j})^T$. For all patients on *j*th gene set, *j* = 1,…, 11, we assume a Cox-ridge model:

$$h_j(t \mid \mathbf{X}_j^{Training}) = h_{0j}(t)\exp(\underline{\beta}_j^T \mathbf{X}_j^{Training}) \quad (5)$$

where $\mathbf{X}_j^{Training}$ is the notation for the training set for j^{th} gene set, which is the gene expression matrix matched on MicMa for j^{th} gene set. Gene set *j* consists a total n_j genes. $\underline{\beta}_j = (\beta_{j1}, ..., \beta_{j,n_j})^T$ are coefficients associated with corresponding genes and $h_{0j}(t)$ is the gene-set specific baseline hazard at certain time point *t*. It has been recommended to standardize the covariates before applying the ridge regression, since the ridge estimates are not invariant to the scaling of the input data [22,27]. In our case, the expression for each gene in a matched gene-set expression matrix was transformed to center around 0 with standard deviation 1. Standardization was carried out both for the training set and the test set.

From *Model 5* on the training data, we obtained the estimated coefficients $\hat{\underline{\beta}}_j$. The *estimated* Prognostic Index for each MicMa patient in training set was calculated by

$$\mathbf{PI}_j^{Estimated} = \hat{\underline{\beta}}_j^T \mathbf{X}_j^{Training} \quad (6)$$

The *predicted* Prognostic Index for an Ull patient in test was calculated by sum of weighted expression for each gene in test dataset by the corresponding coefficient for the same gene estimated from training data:

$$\mathbf{PI}_j^{Predicted} = \hat{\underline{\beta}}_j^T \mathbf{X}_j^{Test} \quad (7)$$

Model evaluation: proportion of variation explained (PVE)

Comparable with the R^2 in regression modeling, the importance of covariates in the Cox model can be quantified using the proportion of variation explained in the outcome variable (PVE) [28] by one or more covariates:

$$R_M^2 = 1 - \left(L_R / L_U\right)^{2/n}$$

L_R : likelihood of a model without covariates (restricted)

L_U : likelihood of a model with covariates (unrestricted)

where n denotes total sample size. The relative importance of a covariate in a multivariate Cox model was measured by the partial PVE, which was calculated as the different of R_M^2 for the full model and R_M^2 for a model with a factor of interest excluded.

Model evaluation: concordance index (C-index)

The concordance index (C-index) [29] is a widely accepted measurement for predictive discrimination of a given model. In survival analysis, it is a generalization of the area under the receiver operating characteristic (ROC) curve, and it measures the probability of concordance between the predicted and observed responses in terms of lengths of survival of any two patients:

$$C - index = \frac{\sum_{i,j \in \Omega} 1\{r_i > r_j\}}{|\Omega|}$$

where r_i and r_j are the predicted risk for ith and jth patient; respectively. Ω is a set of all possible pairs of patients, at least one of whom has experienced an event and time to event $t_i < t_j$. If the predicted risk is larger for the patient who lived shorter, the predictions for that pair are said to be concordant with the outcomes. The C-index is ranging from 0 to 1. The larger C-index, the better is the predictability of a survival model.

V. Supplementary Results:

Evaluation on the training set for systemic relapse as clinical endpoint

The *estimated* Prognostic Index for each MicMa patient in training set was calculated by model 6. It should be emphasized that the results on the training set should not be interpreted as more than a preliminary screening of the model applicability. The performance of the model on the training set was examined as follows. The estimated prognostic index for each training patient was obtained by weighted sum of the expression values of corresponding genes in each gene set in training set (formula 6). Hierarchical clustering using Spearman correlation and average linkage performed on the estimated PIs from all gene sets revealed two distinct risk groups, namely: high-risk and low-risk group (Figure S2A) associated with significantly different survival probabilities (χ^2 = 49.7, df = 1, p < 0.001) (Figure S2B). The median survival time, where half of the subjects have reached the event of interest, was not observed in the low-risk group, while the high-risk group had a 47.2 month median survival time. Distinct clinical characteristics were observed in the two risk clusters: a total of 30 out of 49 Luminal A tumors (61%) were clustered in the low risk group, and 19 luminal A tumors (39%) were found in the high risk group (Table 2). The results suggested that the two risk groups derived from the estimated prognostic indices had distinct survival patterns on the training set.

Table 2. Clinical characteristics for risk groups resulting from hierarchical clustering of estimated PI matrix on training set.

	Low risk	High risk
Number of patients	63	55
Event	4	34
Median survival (month)	-	47.2
LumA (%)	30/49 (61)	19/49 (39)
Basal (%)	9/16 (56)	7/16 (44)

Results summary: analysis for breast cancer specific death as clinical endpoint

1. PCA summary for endpoint breast cancer specific death

Standard deviations:
0.41 0.18 0.11 0.09 0.06 0.06 0.05 0.03 0.02 0.02

Rotation:

	PC1	PC2	PC3	PC4	PC5	PC6	PC7	PC8	PC9	PC10
RS	0.24	-0.18	0.11	-0.16	0.75	0.50	0.08	-0.23	0.07	-0.05
SD	0.10	0.05	0.81	-0.24	-0.32	0.29	-0.27	0.09	0.01	0.02
AMST	0.54	-0.23	0.19	0.78	-0.07	-0.03	0.09	0.01	0.00	0.02
ROT	0.26	0.93	-0.10	0.14	0.07	0.17	-0.06	-0.07	-0.04	0.02
Grade	0.41	-0.10	-0.31	-0.22	0.00	0.15	-0.18	0.78	-0.09	0.00
Robust	0.50	-0.15	-0.32	-0.33	-0.44	0.09	-0.05	-0.54	-0.01	0.10
Hypoxia	0.13	0.10	0.16	-0.22	-0.13	-0.01	0.93	0.15	-0.05	0.00
Stem	0.06	0.04	0.00	-0.04	0.03	-0.10	0.01	0.09	0.89	0.44
Intrinsic	0.35	0.07	0.24	-0.27	0.34	-0.76	-0.12	-0.05	-0.17	0.08
WR	0.12	0.04	0.00	-0.05	-0.06	-0.12	-0.02	-0.01	0.41	-0.89

Importance of components:

	PC1	PC2	PC3	PC4	PC5	PC6	PC7	PC8	PC9	PC10
Standard deviation	0.41	0.18	0.11	0.09	0.06	0.06	0.05	0.03	0.02	0.02
Proportion of Variance	0.73	0.14	0.05	0.04	0.01	0.01	0.01	0.00	0.00	0.00
Cumulative Proportion	0.73	0.87	0.92	0.96	0.97	0.98	0.99	1.00	1.00	1.00

Projected scores of test patients on PCs:

	PC1	PC2	PC3	PC4	PC5	PC6	PC7	PC8	PC9	PC10
ULL_002	-0.17	0.12	-0.15	0.04	0.01	-0.01	0.00	0.03	0.00	0.02
ULL_007	-0.05	-0.04	-0.14	0.02	-0.05	-0.01	0.01	-0.03	0.02	-0.01
ULL_011	0.24	0.29	-0.18	0.09	0.02	0.02	0.01	-0.01	0.00	-0.01
ULL_013	-0.23	0.11	0.19	0.13	0.04	-0.04	0.07	-0.02	0.03	0.02
ULL_014	-0.19	-0.12	-0.07	-0.05	-0.05	-0.01	0.04	0.02	-0.01	0.00
ULL_016	-0.12	-0.02	-0.05	-0.16	0.01	0.02	-0.04	0.02	0.02	0.01
ULL_019	0.03	0.20	-0.12	0.06	0.10	0.09	-0.08	0.04	0.05	0.00
ULL_020	-0.24	0.24	-0.18	-0.02	0.03	-0.01	0.00	0.00	-0.01	-0.01
ULL_022	-0.23	-0.06	-0.25	0.00	0.07	-0.09	-0.06	-0.05	0.01	0.01
ULL_023	0.39	-0.04	-0.09	0.11	0.05	0.10	0.02	-0.02	0.02	-0.03
ULL_024	-0.18	0.14	0.01	0.23	0.01	0.01	-0.08	-0.02	-0.03	0.02
ULL_026	0.57	0.16	0.06	0.00	0.05	0.01	-0.06	0.02	-0.01	0.02
ULL_027	-0.05	-0.11	-0.03	0.04	0.02	-0.08	0.03	-0.02	-0.01	0.00
ULL_028	-0.17	-0.07	-0.18	0.04	0.03	-0.06	-0.01	-0.01	0.01	0.01
ULL_031	-0.35	0.10	0.16	0.03	0.00	-0.02	-0.05	0.01	0.05	0.01
ULL_036	-0.41	-0.15	-0.09	0.11	-0.02	0.06	0.05	-0.03	0.00	0.00
ULL_037	-0.51	-0.13	-0.10	-0.02	0.01	-0.02	0.02	0.03	-0.02	0.00
ULL_038	0.38	-0.24	-0.06	0.08	-0.01	-0.11	0.07	0.04	0.02	0.00
ULL_044	-0.05	-0.36	-0.11	-0.04	-0.01	-0.02	-0.07	0.02	0.01	0.01
ULL_046	-0.06	0.03	0.04	0.02	-0.03	0.08	0.04	-0.04	-0.03	0.02
ULL_048	-0.34	-0.17	-0.04	0.04	-0.01	-0.07	0.03	-0.04	0.01	0.03
ULL_053	0.22	-0.37	-0.06	-0.04	0.06	-0.02	0.06	-0.03	0.00	0.01
ULL_055	-0.75	0.12	0.08	-0.06	0.03	-0.08	0.00	0.01	-0.02	-0.02
ULL_056	0.32	0.29	-0.07	-0.10	-0.07	0.04	0.12	0.00	-0.02	-0.02
ULL_057	0.70	0.21	0.06	-0.11	-0.06	0.00	-0.01	0.00	0.00	0.00
ULL_060	-0.20	0.25	-0.12	-0.15	-0.06	-0.05	-0.01	-0.03	0.02	-0.03
ULL_062	-0.65	0.11	0.28	0.12	-0.04	0.09	0.05	0.03	0.02	-0.01
ULL_063	-0.26	0.25	0.07	-0.03	-0.06	0.02	0.03	0.01	-0.02	0.00

ULL_065	0.95	0.11	0.20	-0.01	-0.09	-0.03	-0.01	0.03	0.02	-0.01
ULL_066	0.30	0.07	-0.06	-0.11	-0.12	0.04	-0.08	-0.01	-0.01	0.00
ULL_067	0.12	-0.55	0.01	-0.04	-0.05	0.04	-0.05	-0.02	0.02	0.00
ULL_069	0.30	0.00	-0.02	0.00	-0.06	0.01	0.08	0.00	-0.03	0.01
ULL_071	0.62	0.09	0.05	-0.08	0.13	0.09	-0.08	-0.04	0.00	-0.01
ULL_072	0.11	0.01	-0.01	-0.06	-0.04	0.03	-0.04	0.00	0.02	-0.01
ULL_074	-0.61	0.08	0.08	-0.11	-0.09	0.00	0.01	-0.03	0.02	0.01
ULL_075	0.25	-0.33	0.00	-0.04	0.08	-0.03	-0.03	0.02	-0.02	0.00
ULL_079	-0.25	0.02	-0.05	0.04	-0.20	-0.01	0.02	-0.02	0.03	0.00
ULL_080	0.39	0.02	-0.04	-0.24	0.05	0.02	-0.04	0.06	0.01	0.01
ULL_083	0.81	-0.15	0.22	0.03	0.05	0.02	0.02	-0.04	0.01	-0.03
ULL_085	-0.15	-0.14	0.07	0.06	0.04	0.02	0.02	-0.03	0.01	0.01
ULL_087	0.23	0.06	-0.05	-0.08	0.05	0.06	0.08	-0.06	0.03	-0.01
ULL_088	-0.23	0.06	0.08	-0.05	0.00	-0.01	0.04	-0.01	0.00	0.04
ULL_096	0.84	-0.14	0.16	-0.02	-0.05	0.04	0.07	-0.02	0.00	0.01
ULL_097	-0.40	0.07	0.02	0.05	0.02	0.00	0.05	-0.01	0.02	0.02
ULL_099	0.38	-0.06	-0.03	-0.03	0.02	0.01	-0.01	0.04	-0.03	0.02
ULL_101	0.40	0.06	0.08	0.09	0.07	-0.12	0.03	0.05	-0.02	-0.01
ULL_105	0.11	-0.03	0.23	0.13	-0.02	0.02	-0.09	-0.01	-0.03	0.01
ULL_107	-0.29	0.04	0.01	0.08	0.01	-0.05	-0.04	0.02	-0.01	-0.02
ULL_111	-0.14	0.04	-0.11	0.02	-0.07	0.11	0.02	0.01	-0.02	-0.03
ULL_112	-0.50	0.14	0.02	0.12	-0.07	0.01	0.05	0.03	-0.02	0.00
ULL_113	0.34	0.04	-0.16	-0.05	-0.01	-0.07	-0.02	0.04	0.01	-0.02
ULL_122	-0.07	0.28	-0.12	0.05	-0.05	-0.06	0.03	-0.02	0.00	0.00
ULL_123	0.15	0.07	-0.06	0.29	0.05	0.06	0.00	0.03	0.00	0.02
ULL_132	0.15	0.02	0.07	0.00	0.01	-0.17	-0.03	-0.01	-0.01	-0.03
ULL_134	-0.28	0.06	0.13	0.04	-0.01	-0.07	-0.08	-0.04	0.00	0.01
ULL_135	-0.27	-0.17	0.13	0.03	0.03	0.03	-0.03	-0.01	0.00	-0.01
ULL_136	-0.42	0.10	0.09	-0.03	0.08	-0.01	0.02	0.04	0.03	0.01
ULL_138	-0.30	-0.44	0.02	-0.04	0.01	0.03	0.04	0.02	-0.03	-0.01
ULL_139	0.33	-0.04	0.06	0.05	0.03	0.04	0.07	0.08	0.01	-0.01
ULL_143	0.12	0.01	-0.09	0.05	-0.01	-0.03	-0.03	0.02	-0.02	0.00
ULL_144	-0.45	0.03	0.11	0.01	0.00	-0.06	-0.02	0.02	0.01	-0.02
ULL_150	-0.35	-0.07	-0.13	0.07	-0.04	0.06	-0.03	0.01	-0.01	-0.02
ULL_165	0.31	-0.01	-0.08	0.06	-0.10	0.01	-0.06	0.02	0.05	0.01
ULL_167	0.63	0.30	-0.05	-0.10	0.05	0.00	0.04	-0.03	-0.02	0.01
ULL_168	-0.36	0.16	-0.04	0.08	0.02	0.04	-0.04	-0.02	0.00	-0.02
ULL_169	0.34	0.06	0.03	-0.12	0.14	0.00	0.06	-0.04	0.02	0.00
ULL_176	-0.37	0.27	0.10	-0.03	0.07	0.02	-0.05	-0.05	-0.03	-0.02
ULL_177	1.03	-0.10	0.12	-0.05	-0.13	-0.10	-0.05	-0.01	0.00	0.01
ULL_181	-0.20	0.24	0.04	0.03	0.01	-0.07	0.02	0.00	0.00	0.01
ULL_183	0.03	-0.27	0.03	-0.04	0.00	0.00	-0.09	-0.02	-0.02	-0.01
ULL_184	0.09	-0.12	0.00	0.04	0.04	-0.02	0.05	-0.01	0.01	-0.02
ULL_188	0.83	0.14	-0.05	0.14	0.02	0.12	-0.05	0.01	-0.01	0.01
ULL_190	-0.25	-0.33	0.04	-0.04	-0.04	0.05	-0.01	0.02	0.01	0.00
ULL_199	-0.40	-0.19	0.01	-0.05	0.02	0.05	-0.01	-0.01	-0.03	0.00
ULL_201	-0.25	0.03	-0.08	-0.18	-0.04	0.12	0.01	0.04	0.01	0.03
ULL_202	0.37	0.06	0.02	-0.05	0.07	-0.03	0.09	0.02	-0.01	0.01
ULL_214	-0.09	0.05	-0.06	-0.05	0.00	-0.01	0.03	-0.01	-0.03	0.03
ULL_216	-0.06	0.22	0.08	-0.05	-0.02	-0.04	-0.06	0.00	-0.02	-0.01
ULL_222	-0.44	-0.31	0.08	0.00	0.00	0.06	0.00	-0.01	-0.01	-0.01
ULL_230	0.12	-0.30	-0.11	0.09	0.03	-0.05	0.03	0.00	0.00	-0.01
DNR_N_AO100	-1.14	0.06	0.15	-0.27	0.08	0.02	-0.01	0.03	0.00	0.00

2. Univariate analysis of Combined PI-risk predictor for BC specific death.

Univariate Cox model incorporating the dichotomized combined-PI as predictor

```
coxph(formula = Surv(time, event == 1) ~ combined_PI, data = tmp)

n= 77, number of events= 30
   (4 observations deleted due to missingness)

                        coef exp(coef) se(coef)     z Pr(>|z|)
combined_PI:high risk 1.2130    3.3636   0.4012 3.024   0.0025 **
---
Signif. codes:  0 '***' 0.001 '**' 0.01 '*' 0.05 '.' 0.1 ' ' 1

                      exp(coef) exp(-coef) lower .95 upper .95
combined_PI:high risk     3.364     0.2973     1.532     7.384

Rsquare= 0.124   (max possible= 0.955 )
Likelihood ratio test= 10.16  on 1 df,   p=0.001436
Wald test            = 9.14  on 1 df,   p=0.002499
Score (logrank) test = 10.26  on 1 df,   p=0.001357
```

Test proportional hazards assumption for individual univariate Cox models reported in the univariate comparison part

```
$combined_PI
                                rho chisq     p
       combined_PI:high risk 0.0766 0.175 0.676
$tumorSize
                            rho  chisq     p
       tumorSizepT2     -0.0793 0.1851 0.667
       tumorSizepT3-pT4 -0.0280 0.0228 0.880
       GLOBAL                NA 0.2558 0.880
$histoGrade
                     rho chisq      p
       histoGrade2 0.343  3.56 0.0593
       histoGrade3 0.233  1.63 0.2012
       GLOBAL         NA  4.78 0.0916
$TP53
                      rho chisq     p
       TP53mutation -0.22  1.33 0.249
$node
                       rho chisq     p
       nodepN1      0.0845 0.209 0.647
       nodepN2-pN3 -0.0775 0.169 0.681
       nodeOther   -0.0781 0.172 0.679
       GLOBAL           NA 0.718 0.869
$ER
                   rho chisq      p
       ERpositive 0.495  6.32 0.0119
$stage
                 rho  chisq     p
       stage2 -0.0548 0.0743 0.785
       stage3 -0.0696 0.1184 0.731
       stage4  0.1268 0.4121 0.521
       GLOBAL      NA 1.7342 0.629
$AOL_risk
```

```
                rho chisq     p
AOL_risk 0.0565 0.075 0.784
```
$RS
```
           rho chisq    p
RS1 -0.164  0.77 0.38
```
$SD
```
           rho chisq      p
SD1 -0.423  5.12 0.0236
```
$AMST
```
             rho  chisq     p
AMST1 0.0306 0.0277 0.868
```
$ROT
```
            rho chisq     p
ROT1 -0.213  1.29 0.257
```
$Grade
```
               rho    chisq     p
Grade1 0.00343 0.000345 0.985
```
$Robust
```
                rho chisq     p
Robust1 -0.0733 0.156 0.693
```
$Hypoxia
```
                rho chisq      p
Hypoxia1 -0.347  3.38 0.0659
```
$Stem
```
             rho chisq     p
Stem1 -0.136 0.529 0.467
```
$Intrinsic
```
                  rho chisq     p
Intrinsic1 0.0509 0.076 0.783
```
$WR
```
            rho chisq     p
WR1 -0.0809 0.188 0.664
```

Summary of univariate comparison

	Deviance	PVE	p	Cindex	HR	HR_lowerCI	HR_upperCI	type
tumorSize	9.706	0.121	0.008	0.735	NA	NA	NA	Clinical parameter
histoGrade	0.759	0.010	0.684	0.539	NA	NA	NA	Clinical parameter
TP53	9.643	0.118	0.002	0.801	3.460	1.661	7.210	Clinical parameter
node	10.266	0.125	0.016	0.603	NA	NA	NA	Clinical parameter
ER	0.510	0.007	0.475	0.373	0.753	0.349	1.626	Clinical parameter
stage	4.927	0.074	0.177	0.670	NA	NA	NA	Clinical parameter
AOL_risk	1.417	0.020	0.234	0.684	2.198	0.517	9.353	Clinical parameter
RS	5.822	0.073	0.016	0.735	2.473	1.155	5.293	GeneSet predictor
SD	0.741	0.010	0.389	0.634	1.380	0.657	2.903	GeneSet predictor
AMST	9.307	0.114	0.002	0.763	3.188	1.455	6.986	GeneSet predictor
ROT	1.361	0.018	0.243	0.636	1.534	0.744	3.162	GeneSet predictor
Grade	6.625	0.082	0.010	0.730	2.629	1.226	5.636	GeneSet predictor
Robust	4.341	0.055	0.037	0.698	2.166	1.030	4.555	GeneSet predictor
Hypoxia	3.208	0.041	0.073	0.702	1.929	0.936	3.977	GeneSet predictor
Stem	0.172	0.002	0.679	0.557	1.164	0.567	2.389	GeneSet predictor
Intrinsic	4.510	0.057	0.034	0.687	2.199	1.045	4.625	GeneSet predictor
WR	4.369	0.055	0.037	0.699	2.161	1.039	4.497	GeneSet predictor
combined_PI	10.159	0.124	0.001	0.768	3.364	1.532	7.384	GeneSet predictor

3. Multivariate analysis of Combined PI-risk predictor for BC death.

Full model: Risk = tumor size + node + TP53 + combined_PI + strata(ER)

```
                        coef exp(coef) se(coef)     z     p
tumorSize:pT2          0.766     2.151    0.665  1.15 0.250
tumorSize:pT3-pT4      1.534     4.639    0.824  1.86 0.062
node:pN1              -1.068     0.344    0.672 -1.59 0.110
node:pN2-pN3           1.323     3.754    0.595  2.22 0.026
node:Other             1.460     4.307    0.602  2.43 0.015
TP53:mutation          1.436     4.202    0.697  2.06 0.039
combined_PI:high risk  1.214     3.366    0.575  2.11 0.035

Likelihood ratio test=36.2  on 7 df, p=6.57e-06  n= 68, number of events= 27
   (13 observations deleted due to missingness)
```

Correlation between TP53 & combined_PI predictor

```
      low risk high risk
  WT        39        21
  Mut        1        19

       Fisher's Exact Test for Count Data
p-value = 3.049e-06
alternative hypothesis: true odds ratio is not equal to 1
95 percent confidence interval:
    4.751341 1490.362021
sample estimates:
odds ratio
  33.88675
```

Model comparison by AIC and Analysis of Deviance:
Full model vs Model 2 [Risk = tumor size + node + TP53 + strata(ER)]

```
>  stepAIC(model2)
Start:  AIC=152.41
Surv(time, event == 1) ~ tumorSize + node + TP53 + strata(ER)

> stepAIC(model_full)
Start:  AIC=149.96
Surv(time, event == 1) ~ tumorSize + node + TP53 + combined_PI + strata(ER)

Analysis of Deviance Table
 Cox model: response is  Surv(time, event == 1)
 Model 1: ~ tumorSize + node + TP53 + combined_PI + strata(ER)
 Model 2: ~ tumorSize + node + TP53 + strata(ER)
   loglik  Chisq Df P(>|Chi|)
1 -67.982
2 -70.203 4.4426  1   0.03505 *
---
Signif. codes:  0 '***' 0.001 '**' 0.01 '*' 0.05 '.' 0.1 ' ' 1
```

Model selection by AIC:

```
> stepAIC(model_full, direction = "both")
Start:  AIC=149.96
Surv(time, event == 1) ~ tumorSize + node + TP53 + combined_PI + strata(ER)
              Df    AIC
```

```
- tumorSize    2 149.67
<none>           149.96
- combined_PI  1 152.41
- TP53         1 152.85
- node         3 159.63

Step:  AIC=149.67
Surv(time, event == 1) ~ node + TP53 + combined_PI + strata(ER)
              Df    AIC
<none>           149.67
+ tumorSize    2 149.96
- TP53         1 150.68
- combined_PI  1 155.25
- node         3 158.79
```

Final model: Risk = node + TP53 + combined_PI + strata(ER)

```
  n= 68, number of events= 27

                        coef exp(coef) se(coef)      z Pr(>|z|)
node:pN1             -0.8592    0.4235   0.6520 -1.318  0.18756
node:pN2-pN3          1.5807    4.8586   0.5757  2.746  0.00603 **
node:Other            1.3127    3.7161   0.5848  2.245  0.02479 *
TP53:mutation         0.9547    2.5980   0.5593  1.707  0.08783 .
combined_PI:high risk  1.5307    4.6215   0.5487  2.790  0.00527 **
---
Signif. codes:  0 '***' 0.001 '**' 0.01 '*' 0.05 '.' 0.1 ' ' 1

                     exp(coef) exp(-coef) lower .95 upper .95
node:pN1                0.4235     2.3613     0.118     1.520
node:pN2-pN3            4.8586     0.2058     1.572    15.015
node:Other              3.7161     0.2691     1.181    11.691
TP53:mutation           2.5980     0.3849     0.868     7.776
combined_PI:high risk    4.6215     0.2164     1.577    13.546

Rsquare= 0.38   (max possible= 0.921)
Likelihood ratio test= 32.52  on 5 df,   p=4.697e-06
Wald test            = 27.56  on 5 df,   p=4.432e-05
Score (logrank) test = 32.92  on 5 df,   p=3.907e-06
```

Test proportional hazards assumption on the final model

```
                         rho  chisq      p
node:pN1              0.3320 3.0558 0.0804
node:pN2-pN3          0.1685 0.8135 0.3671
node:Other           -0.0485 0.0725 0.7878
TP53:mutation         0.0460 0.0421 0.8374
combined_PI:high risk  0.2846 2.9546 0.0856
GLOBAL                    NA 7.6262 0.1781
```

Supplementary References

1. Naume B, Zhao X, Synnestvedt M, Borgen E, Russnes H, et al. (2007) Presence of bone marrow micrometastasis is associated with different recurrence risk within molecular subtypes of breast cancer. Molecular Oncology 1: 160-171.
2. Wiedswang G, Borgen E, Kåresen R, Kvalheim G, Nesland J, et al. (2003) Detection of isolated tumor cells in bone marrow is an independent prognostic factor in breast cancer. Journal of Clinical Oncology 21: 3469.
3. Zhao H, Hastie T, Whitfield ML, Borresen-Dale AL, Jeffrey SS (2002) Optimization and evaluation of T7 based RNA linear amplification protocols for cDNA microarray analysis. BMC Genomics 3: 31.
4. Langerød A, Zhao H, Borgan O, Nesland J, Bukholm I, et al. (2007) TP53 mutation status and gene expression profiles are powerful prognostic markers of breast cancer. Breast Cancer Res 9: R30.
5. Paik S, Shak S, Tang G, Kim C, Baker J, et al. (2004) A multigene assay to predict recurrence of tamoxifen-treated, node-negative breast cancer. N Engl J Med 351: 2817-2826.
6. Finak G, Bertos N, Pepin F, Sadekova S, Souleimanova M, et al. (2008) Stromal gene expression predicts clinical outcome in breast cancer. Nat Med 14: 518-527.
7. Minn AJ, Gupta GP, Siegel PM, Bos PD, Shu W, et al. (2005) Genes that mediate breast cancer metastasis to lung. Nature 436: 518-524.
8. van 't Veer LJ, Dai H, van de Vijver MJ, He YD, Hart AA, et al. (2002) Gene expression profiling predicts clinical outcome of breast cancer. Nature 415: 530-536.
9. Mook S, Schmidt MK, Viale G, Pruneri G, Eekhout I, et al. (2008) The 70-gene prognosis-signature predicts disease outcome in breast cancer patients with 1-3 positive lymph nodes in an independent validation study. Breast Cancer Res Treat.
10. Wang Y, Klijn JG, Zhang Y, Sieuwerts AM, Look MP, et al. (2005) Gene-expression profiles to predict distant metastasis of lymph-node-negative primary breast cancer. Lancet 365: 671-679.
11. Sotiriou C, Wirapati P, Loi S, Harris A, Fox S, et al. (2006) Gene expression profiling in breast cancer: understanding the molecular basis of histologic grade to improve prognosis. J Natl Cancer Inst 98: 262-272.
12. van Vliet MH, Reyal F, Horlings HM, van de Vijver MJ, Reinders MJ, et al. (2008) Pooling breast cancer datasets has a synergetic effect on classification performance and improves signature stability. BMC Genomics 9: 375.
13. Chi JT, Wang Z, Nuyten DS, Rodriguez EH, Schaner ME, et al. (2006) Gene expression programs in response to hypoxia: cell type specificity and prognostic significance in human cancers. PLoS Med 3: e47.
14. Liu R, Wang X, Chen GY, Dalerba P, Gurney A, et al. (2007) The prognostic role of a gene signature from tumorigenic breast-cancer cells. N Engl J Med 356: 217-226.
15. Perou CM, Sorlie T, Eisen MB, van de Rijn M, Jeffrey SS, et al. (2000) Molecular portraits of human breast tumours. Nature 406: 747-752.
16. Sørlie T, Perou CM, Tibshirani R, Aas T, Geisler S, et al. (2001) Gene expression patterns of breast carcinomas distinguish tumor subclasses with clinical implications. Proc Natl Acad Sci U S A 98: 10869-10874.
17. Sørlie T, Tibshirani R, Parker J, Hastie T, Marron JS, et al. (2003) Repeated observation of breast tumor subtypes in independent gene expression data sets. Proc Natl Acad Sci U S A 100: 8418-8423.
18. Hu Z, Fan C, Oh DS, Marron JS, He X, et al. (2006) The molecular portraits of breast tumors are conserved across microarray platforms. BMC Genomics 7: 96.
19. Chang HY, Sneddon JB, Alizadeh AA, Sood R, West RB, et al. (2004) Gene expression signature of fibroblast serum response predicts human cancer progression: similarities between tumors and wounds. PLoS Biol 2: E7.

20. Ravdin P, Siminoff L, Davis G, Mercer M, Hewlett J, et al. (2001) Computer program to assist in making decisions about adjuvant therapy for women with early breast cancer. Journal of Clinical Oncology 19: 980.
21. Cox D (1972) Regression models and life-tables. Journal of the Royal Statistical Society Series B (Methodological) 34: 187-220.
22. Frank I, Friedman J (1993) A statistical view of some chemometrics regression tools. Technometrics 35: 109-135.
23. Verweij PJ, Van Houwelingen HC (1994) Penalized likelihood in Cox regression. Stat Med 13: 2427-2436.
24. Verweij PJ, Van Houwelingen HC (1993) Cross-validation in survival analysis. Stat Med 12: 2305-2314.
25. Van Houwelingen JC, Le Cessie S (1990) Predictive value of statistical models. Stat Med 9: 1303-1325.
26. Bøvelstad HM, Nygard S, Størvold HL, Aldrin M, Borgan O, et al. (2007) Predicting survival from microarray data--a comparative study. Bioinformatics 23: 2080-2087.
27. Marquardt D (1980) Comment on a critique of some ridge regression methods. J Amer Statist Assoc 75: 87-91.
28. Schemper M (1993) The relative importance of prognostic factors in studies of survival. Statistics in medicine 12: 2377-2382.
29. Harrell F, Lee K, Mark D (1996) Tutorial in biostatistics multivariable prognostic models: issues in developing models, evaluating assumptions and adequacy, and measuring and reducing errors. Statistics in medicine 15: 361-387.
